# Horizontal gene transfer in chromalveolates

**DOI:** 10.1186/1471-2148-7-173

**Published:** 2007-09-25

**Authors:** Tetyana Nosenko, Debashish Bhattacharya

**Affiliations:** 1 University of Iowa, Department of Biological Sciences and the Roy J. Carver Center for Comparative Genomics, 446 Biology Building, Iowa City, Iowa 52242, USA

## Abstract

**Background:**

Horizontal gene transfer (HGT), the non-genealogical transfer of genetic material between different organisms, is considered a potentially important mechanism of genome evolution in eukaryotes. Using phylogenomic analyses of expressed sequence tag (EST) data generated from a clonal cell line of a free living dinoflagellate alga *Karenia brevis*, we investigated the impact of HGT on genome evolution in unicellular chromalveolate protists.

**Results:**

We identified 16 proteins that have originated in chromalveolates through ancient HGTs before the divergence of the genera *Karenia *and *Karlodinium *and one protein that was derived through a more recent HGT. Detailed analysis of the phylogeny and distribution of identified proteins demonstrates that eight have resulted from independent HGTs in several eukaryotic lineages.

**Conclusion:**

Recurring intra- and interdomain gene exchange provides an important source of genetic novelty not only in parasitic taxa as previously demonstrated but as we show here, also in free-living protists. Investigating the tempo and mode of evolution of horizontally transferred genes in protists will therefore advance our understanding of mechanisms of adaptation in eukaryotes.

## Background

Horizontal gene transfer (HGT) is the movement of genetic material between different species and is considered to be one of the major driving forces of prokaryotic evolution [1-4]. Until recently, it was believed that this phenomenon was largely restricted to the prokaryotic domain. In eukaryotes, gene duplication has classically been viewed as the major source of genetic novelty [5,6]; this paradigm of eukaryotic evolution is based on genome studies of model organisms such as multicellular plants, animals, and fungi. In the last decade, rapid accumulation of genome data from unicellular eukaryotes, protists, has allowed researchers to reassess the role of HGT in eukaryotic evolution. The results of comparative analyses of genomes of anaerobic parasitic protists provided a major breakthrough in our understanding of the impact of interdomain HGT in eukaryotes. For example, 96 potential cases of prokaryote-to-eukaryote HGT were identified in the genome of an intestinal parasite of humans and animals *Entamoeba histolytica *[7], 84 in the fish parasite *Spironucleus salmonicida *[8], 152 in a sexually transmitted human pathogen *Trichomonas vaginalis *[9], 24 in *Cryptosporidium parvum *[10], and 148 in anaerobic rumen ciliates [11]. These numbers comprise up to 4% of genes in the extremely reduced genomes of these anaerobic protists. It is believed that the acquisition of bacterial genes by these eukaryotes accelerated their adaptation to anaerobic environments and the transition to a parasitic life style. Several recent reports indicate that HGT also plays a role in the genome evolution of free-living protists. Analysis of the complete genome sequence of the soil amoeba *Dictyostelium discoideum *led to the identification of 18 genes derived from prokaryotes [12]. Several cases of HGT have been reported for dinoflagellate and chlorarachniophyte algae [13-16]. The fact that complete genome sequences are available now for a limited number of free living protists explains a significant disproportion in the study of HGT in different groups of protists. However, public databases also contain Expressed Sequence Tag (EST) libraries for over 50 species of free living unicellular eukaryotes [17,18] that can also be used to assess the impact of HGT on genome evolution in protists.

Here we analyze EST and complete genome data to study HGT in chromalveolate protists. Chromalveolates comprise the six eukaryotic lineages, cryptophytes, haptophytes, stramenopiles, ciliates, apicomplexans, and dinoflagellates and have adapted to a wide variety of environments. They are characterized by a tremendous diversity of forms and modes of nutrition including heterotrophy, parasitism, phototrophy, and mixotrophy. According to the chromalveolate hypothesis, the common ancestor of the six constituent lineages was a free living photosynthetic organism that derived its plastid *via *a red algal secondary endosymbiosis [19]. Within-chromalveolate taxon relationships and the monophyly of this group are controversial [20]. Nuclear gene phylogenies support the monophyly of stramenopiles, ciliates, apicomplexans, and dinoflagellates and monophyly of cryptophytes and haptophytes [21,22]. However, relationships between the two clades still remain unresolved.

Gene movement from the endosymbiont to the host nucleus is a specific instance of HGT that is referred to as endosymbiotic gene transfer (EGT). The impact of EGT on the evolution of chromalveolate genomes has been intensively studied in the last decade [23-27] and will not be considered here. We limited our research to gene transfers from non-organellar sources. To identify genes acquired by chromalveolates through HGT at different time points in their evolutionary history, we performed a broad scale phylogenetic analysis of the EST data generated for a free living phototrophic dinoflagellate alga *Karenia brevis *that is renowned as an agent of toxic algal blooms that annually cause massive fish and marine mammal mortality in the Gulf of Mexico [28]. Detailed analyses of the identified genes presented in this paper suggest that recurring inter- and intradomain gene movement should be considered as an important source of genetic novelty in chromalveolates.

## Results

In this study, we used a combination of four different approaches to identify genes acquired by chromalveolates through HGT (see Methods). The major goal of this study was to discover genes uniquely present in chromalveolates and bacteria. This study is based on the assumption that HGT is the most plausible explanation for the occurrence of bacterial genes in a single eukaryotic lineage. An alternative explanation is that these bacterial genes were derived via intracellular transfer from the mitochondrial progenitor by the ancestral eukaryote and subsequently lost from most taxa. Apart from invoking independent gene losses from potentially many eukaryotic lineages, the latter scenario implies (improbably) that the genome size of the eukaryotic ancestor was far larger than in extant taxa.

Our data screening approach was designed to retrieve relatively ancient cases of HGT that occurred before the divergence of two closely related genera of dinoflagellate algae, *Karenia *and *Karlodinium*. Using a sequence similarity search (BLAST; e-value ≤ 10^-10^) we identified 3,341 genes shared by *K. brevis *and at least 1/5 dinoflagellate species for which EST data are available [18]. To identify genes acquired by chromalveolates through interdomain HGT, we analyzed the restricted set of *K. brevis *genes using a combination of a standard "Best Hit" approach and high throughput automated and manual phylogenomic analyses (see Methods). These analyses yielded 80 unique genes encoding proteins from 45 different families putatively derived from prokaryotes at different time points of chromalveolate evolution. Throughout the text, we will use the term "protein" to unite members of one protein family encoded by distinct genes. Three proteins represented by five unigenes resulted from an additional analysis aimed at detecting *K. brevis *homologs of bacterial proteins involved in cell wall biogenesis (see Methods). Detailed phylogenetic analyses of the identified proteins provide strong support for the origin through HGT of 16 proteins represented by 36 unique genes (Table 1). Six of these proteins are uniquely shared by dinoflagellates and prokaryotes (BLAST; e-value ≤ 10^-20^); two proteins, malate-quinone oxidoreductase and monomeric NADPH-dependent isocitrate dehydrogenase, are present only in several chromalveolate lineages and prokaryotes. Homologs of eight proteins (e-value ≤ 10^-20^) are present in prokaryotes, chromalveolates, and at least one other eukaryotic lineage. Phylogenies of the remaining 32 proteins represented by 49 *K. brevis *EST contigs could not be clarified due to the presence of multiple, highly divergent sequences in different protist lineages that might have resulted either from independent gene transfers or from ancient gene duplications.

**Table 1:** Horizontal gene transfers from bacteria to chromalveolates

**1**	**2**	**3**	**4**	**5**	**6**
**Domain (Pfam)**	**Protein family [Function]**	**Accession number**	**Number unigenes**	**Best Hit**	**Phylogeny**
MVIM	Predicted dehydrogenase	EF540335	7	1e-57	Fig. 2
WECE	Pyridoxal phosphate dependent aminotransferase [Cell envelope biogenesis, outer membrane]	EF540335 EF540337	12	2e-100	Fig. 2
Epimerase	NAD dependent epimerase/dehydratase [Cell envelope biogenesis, outer membrane]	EF540339	2	1e-56	Fig. 3
CAS-like	Clavaminic acid synthetase [Biosynthesis of clavulanic acid]	EF540323 EF540325	3	1e-53	Fig. 4
MQO	Malate-quinone oxidoreductase [Energy metabolism]	EF540331 EF540333	2	1e-98	Fig. 5
NADP-IDH	Monomeric NADP(+)-dependent isocitrate dehydrogenase. [Energy metabolism]	EF540327 EF540328	2	1e-162	Fig. 6
Fe-ADH	Iron-containing alcohol dehydrogenase [Energy metabolism]	EF540326	1	2e-97	Additional file 1
PutA	NAD-dependent aldehyde dehydrogenases [Energy metabolism]	EF540338	2	2e-120	Additional file 1
PBPb	Substrate-bound, membrane-associated, periplasmic binding protein [Substrate transport]	EF540334	1	2e-26	Additional file 1
SIR2	Silent information regulator 2 [Gene silencing, DNA repair]	EF540336	2	5e-39	Additional file 1
AslA	Arylsulfatase A [Substrate transport]	EF540322	1	1e-70	Additional file 1*
COG3129	SAM-dependent methyltransferase	EF540332	1	1e-23	Additional file 1*
ATS1	Alpha-tubulin suppressor [Cell division and chromosome partitioning, cytoskeleton]	EF540324	5	8e-52	Additional file 1*
PdxA	Pyridoxal phosphate biosynthetic protein PdxA [Amino acid metabolism]	EF540340	1	4e-54	Additional file 1*
COG3618	Metal-dependent hydrolase of the TIM-barrel fold	EF540329	2	1e-55	Additional file 1*
COG3022	Hypothetical [unknown]	EF540330	1	4e-26	Additional file 1*

**1**. The Pfam domain designation [29, 30]. **2**. Confirmed or proposed function of the prokaryotic homologs is given. **3**. GenBank accession number. **4**. Numbers of unigenes encoding particular protein identified in the *Karenia brevis *EST data are given. Only one copy of gene was submitted into the GenBank. All gene copies can be found at [111]. **5**. E-values for the best bacterial hit from the GenBank nonredundant database. **6**. Phylogenetic trees for all proteins present in dinoflagellates and at least one other eukaryotic lineage (BLASTp e-value ≤ 10^-10^).

*The protein was found only in dinoflagellates and prokaryotes.

Below we provide a detailed description of the most interesting cases of prokaryote-to-eukaryote HGTs. The direction of interdomain HGTs was inferred based on the relative distribution of the gene among bacteria and eukaryotes. Genes widespread among prokaryotes and rare among eukaryotes were considered to be derived from a prokaryotic donor. The identified proteins are classified in various functional groups based on known functions of their bacterial homologs including plasma membrane biogenesis and biosynthesis of secondary metabolites, energy and amino acid metabolism, substrate transport, regulation of gene expression and DNA repair. In addition, we present results of phylogenetic analyses of translation elongation factor EF2 that represents the only identified case of a recent transfer that occurred after the *Karenia *and *Karlodinium *divergence and the only example of HGT involving a gene of eukaryotic origin.

### Plasma membrane biogenesis and biosynthesis of secondary metabolites

#### Dehydrogenase MVIM-sugar aminotransferase fusion protein

**Figure 1:**
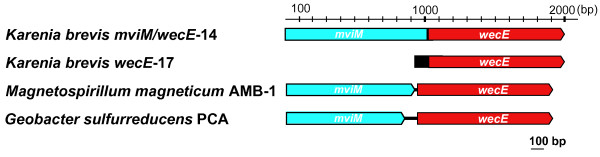
**Structure of the *mviM/wecE *fragment in the dinoflagellate *Karenia brevis *and proteobacteria**. Each arrow-shaped box represents an open reading frame (ORF). Arrows indicate the direction of transcription. Solid black boxes represent 5' untranslated regions (UTR) and protein spacer regions. Black lines connecting two boxes represent intergenic regions.

Screening of the *K. brevis *EST data resulted in the identification of two related proteins, sugar aminotransferase (WECE) and the fused dehydrogenase MVIM-sugar aminotransferase (MVIV-WECE) that showed high similarity to bacterial proteins. Both proteins are encoded by multiple gene copies each represented by multiple transcripts in the *K. brevis *EST data. To simplify matters, we named allMVIV-WECE-encoding genes *mvi*M/*wecE*-14 and all WECE-encoding genes *wecE*-17. The gene names were derived from the names of the corresponding protein domains followed by the number of the contigs encoding the full-length cDNA sequence of these proteins. Bacterial homologs of the *wecE*-17 encode a sugar aminotransferase that is classified in the DegT/DnrJ/EryC1/StrS aminotransferase family in the Pfam database [29,30]. We identified five genes of the *wecE*-17 type in the *K. brevis *genome. The *wecE*-17 genes share 93–99% amino acid sequence identity over their protein coding regions and significant similarity of the 3' UTR sequences. The total length of the identified *wecE*-17 sequences is 442 aa including 367 aa of the mature WECE protein and 74 aa of the incomplete N-terminal extension (Fig. 1). The N-terminus is highly hydrophobic and, according to the protein topology prediction program TMHMM [31], contains a transmembrane motif (P = 0.87%).

The *mviM/wecE*-14 genes have a bipartite structure (Fig. 1). The N-terminal region of this sequence contains a NAD-binding Rossmann fold domain typical for the GFO/IDH/MocA oxidoreductase family and shows high similarity to the dehydrogenase MVIM found in Bacteria and Archaea (55% amino acid sequence identity). The C-terminal domain of *mviM/wecE*-14 encodes WECE that shares 81–84% amino acid sequence identity with protein encoded by *wecE*-17 and about 67% amino acid sequence identity with its bacterial homologs. Using analyses of nucleotide differences in protein coding regions and insertion-deletions in 5' and 3' UTR, we identified at least seven MVIM-WECE-encoding genes in *K. brevis*. Because these genes share 92–99% amino acid sequence identity and retain significant sequence similarity of their 5' and 3' UTRs their origin is likely through recent gene duplications. The *K. brevis *culture that was used for the EST data collection was a vegetative haploid clonal cell line therefore all sequence variants were non-allelic gene copies.

**Figure 2:**
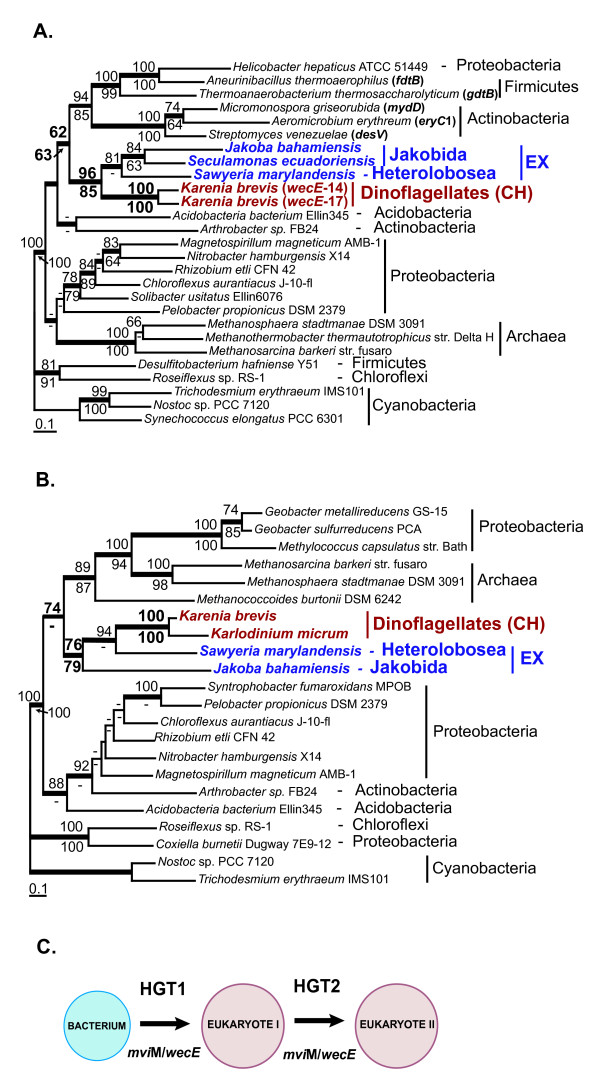
**Origin of sugar aminotransferase WECE and dehydrogenase MVIM in eukaryotes**. **A**. ML tree of sugar aminotransferase **WECE**. **B**. ML tree of dehydrogenase MVIM. The numbers above and below the branches are the results of ML and NJ bootstrap analyses, respectively. Only bootstrap values ≥ 60% are shown. The thick branches indicate ≥ 0.95 posterior probability from a Bayesian inference. Branch lengths are proportional to the number of substitutions per site (see scale bars). Numbers in bold indicate bootstrap support for the monophyly of *wecE*-14 and *wecE*-17 sequences in *Karenia brevis*. CH indicates chromalveolates and EX indicates Excavata. Names of bacterial WECE-encoding genes that have been studied experimentally [34-36, 41, 43] are given in brackets. **C**. Origin and distribution of MVIM and WECE in eukaryotes through sequential HGTs.

The sequence structure is conserved between all *mviM/wecE*-14 copies: they are composed of 361 aa of MVIM, 368 aa of WECE, and 10 aa of the spacer region separated the two domains. *MviM/wecE*-14 genes do not have an N-terminal extension and, presumably, encode cytosolic proteins. The comparison of the *wecE*-14 and *wecE*-17 sequences shows that the two types of transcripts do not result from alternative splicing, but are encoded by different loci in the *K. brevis *genome. The number of amino acid substitutions between the protein coding regions of *wecE*-14 and *wecE*-17 is higher than the number of within group substitutions. Furthermore, the N-terminus and 3' UTRs are highly conserved between *wecE*-17 genes and share no sequence similarity with *mviM/wecE*-14. Phylogenetic analyses provide strong support for *wecE*-14 and *wecE*-17 monophyly (bootstrap proportions, maximum likelihood, BPml = 100%; neighbor joining, BPnj = 100%; Bayesian posterior probability, BPP = 1.0; Fig. 2A).

Homologs of the *K. brevis *MVIM and WECE are broadly distributed among Bacteria and Archaea. Interestingly, the two genes are located in one operon separated by 9–89 bp spacer regions in several proteobacteria (Fig. 1). This observation suggests that dinoflagellates might have derived the *mviM*/*wecE*-14 fragment through a single HGT. *WecE*-17 most likely resulted from a recombination between *MviM/wecE*-14 and a DNA fragment that gave rise to the hydrophobic N-terminus of *wecE*-17. This event occurred after the *Karenia *divergence and was followed by multiple duplications of *wecE*-17 and *mviM*/*wecE*-14. Homologs of the bacterial MVIM and WECE proteins have not previously been reported for eukaryotes. Using a BLAST search against the GenBank dbEST database we identified homologs of the *K. brevis *WECE proteins in free living heterotrophic species of excavates: jakobids *Seculamonas ecuadoriensis *and *Jakoba bahamiensis *and heterolobosean amoebae *Sawyeria marylandensis *(see Additional file 1). Homologs of the *K. brevis *MVIM have been identified in *Karlodinium micrum *and two species of excavates, *J. bahamiensis *and *S. marylandensis *(see Additional file 1). The MVIM and WECE distribution and phylogeny indicate that these proteins have been acquired by excavates before the divergence of jakobids and heteroloboseans (Fig. 2). The observed monophyly of the *K. brevis *and *K. micrum *MVIM sequences and the absence of MVIM and WECE homologs in EST libraries from four species of peridinin dinoflagellates that are ancestral to *Karenia *and *Karlodinium *[32], suggests that the *mvi*M/*wecE*-14 fragment originated on the branch uniting *Karenia *and *Karlodinium*. The result of the MVIM and WECE nucleotide composition analysis also demonstrates that the acquisition of these genes by dinoflagellates and excavates was a relatively ancient event(s). The nuclear gene nucleotide composition varies significantly among different species of excavates and dinoflagellates. However, the GC content of the MVIM- and WECE-encoding sequences does not deviate from the GC content range identified for protein-coding regions in the host organisms (Table 2). Phylogenetic analyses of WECE and MVIM support the monophyly of these sequences in eukaryotes (BPml = 96%; BPnj = 85%; BPP = 1.0 for WECE and BPml = 76%; BPnj = 79%; BPP = 0.97 for MVIM) (Fig. 2A, B). Although we cannot exclude the possibility that dinoflagellates and excavates independently derived MVIM- and WECE-encoding genes from the same bacterial source, the most plausible interpretation of the similar phylogenies for the two genes and the co-occurrence of both sequences in two distantly related groups of eukaryotes is that the bacterial *mviM*/*wecE *DNA fragment was acquired by one of these eukaryotic lineages and passed to the next *via *HGT, perhaps through phagocytosis (Fig. 2C). The incongruence of the prokaryotic MVIM and WECE phylogenies with the respective species phylogenies and disagreement between the MVIM and WECE tree topologies (Fig. 2A, B) do not allow us to unambiguously identify the prokaryotic donor of the *mviM/wecE *fragment. The disagreement between phylogenies may result from frequent transfers among bacteria that has abolished a specific sister group relationship to the eukaryotic sequences (see Discussion for details).

**Table 2:** Nucleotide composition of selected genes acquired by protists through HGT

Species	GC content (%)								
	Genome		Gene^1^						
	average	range	WECE	MVIM	Epimerase	CAS-like	MQO	IDH	EF2
*Karenia brevis*	50.86	45–56	51.84	50.59	53.09	54.44	53.92	53.27	65.16
*Karlodinium micrum*	49.50	44–54	-	49.30	-	51.50	49.35	49.60	*51.94*
*Alexandrium tamarense*	58.93	50–67	-	-	-	67.84	60.75	59.26	*57.92*
*Amphidinium carterae*	54.64	50–58	-	-	-	-	-	53.75	*56.03*
*Heterocapsa triquetra*	63.64	57–69	-	-	-	-	63.64	63.90	*62.93*
*Lingulodinium polyedrum*	62.98	55–69	-	-	-	-	-	57.45	*66.78*
*Phaeodactylum tricornutum*	53.86	49–60	-	-	-	-	-	53.40	*53.19*
*Phytophthora ramorum*	61.15	54–67	-	-	60.65	-	-	-	*62.59*
*Thalassiosira pseudonana*	49.67	45–54	-	-	46.64	-	-	49.91	*50.78*
*Isochrysis galbana*	61.38	54–67	-	-	-	67.41	-	58.51	*64.31*
*Emiliania huxleyi*	67.75	63–72	-	-	-	69.96	62.64	-	-
*Pavlova lutheri*	64.36	59–66	-	-	-	-	-	-	*64.48*
*Giardia lamblia*	52.11	44–63	-	-	52.16	-	-	-	*56.90*
*Spironucleus barkhanus*	43.02	31–63	-	-	52.88	-	-	-	*42.72*
*Jakoba bahamiensis*	60.70	56–62	61.39	60.54	-	-	-	-	*60.54*
*Seculamonas ecuadoriensis*	61.63	56–64	61.66	-	-	-	-	-	*62.53*
*Sawyeria marylandensis*	32.00	22–41	30.51	24.36	-	-	-	-	*30.10*
*Euglena gracilis*	56.53	45–68	-	-	-	-	-	-	*51.12*
*Leishmania major*	60.95	55–64	-	-	-	-	-	-	*63.08*
*Trypanosoma brucei*	54.53	42–59	-	-	-	-	-	-	*53.21*

Nucleotide composition is given for six out of 16 bacteria-derived genes listed in Table 1. The GC content of all bacteria-derived genes identified in *Karenia brevis *does not deviate significantly from the average. The underlined numbers indicate a gene GC content that significantly deviates from the genome GC content. Dashes indicate that the gene have not been found for this species; numbers in italic indicate GC contents for the genes that have a phylogeny consistent with the species phylogeny.

Study of HGT in bacteria demonstrates that genes encoding physiologically coupled reactions are often co-transferred, frequently in operons [33]. The fact that MVIM- and WECE-encoding genes are fused in *K. brevis *and linked in several proteobacteria may indicate that proteins encoded by these genes are functionally coupled. Functions of the dehydrogenase MVIM are poorly characterized in bacteria. The bacterial homologs of WECE have been intensively studied for their involvement in the biosynthesis of microlide antibiotics that belong to the large family of secondary metabolites known as polyketides [34-37], outer membrane liposaccharides [38-40], and surface layer glycoproteins [41-43]. According to the results of phylogenetic analyses, WECE proteinsidentified in eukaryotes form a monophyletic clade with bacterial proteins from two distinct groups (Fig. 2A). The first group is represented by actinobacterial proteins involved in the biosynthesis of microlide antibiotics such as narbomycin, erythromycin, pikromycin, and neomethymycin (Fig. 2A). These catalyze the biosynthesis of the deoxy sugar D-desosamine, the addition of which to the actinobacterial polyketides is crucial for their antibiotic activity [34]. The second group includes monofunctional sugar aminotransferases involved in the glycosylation of the surface layer proteins in firmicutes *Aneurinibacillus thermoaerophilus *(*ftd*B) and *Thermoanaerobacterium thermosaccharolyticum *(*qdt*B). *Ftd*B and *qdt*B encode a key enzyme of the biosynthesis of thymidine diphosphate-activated 3-acetamido-3,6-dideoxy- D- galactose (dTDP-D-Fuc *p*3NAc), which serves as a precursor for the assembly of structural polysaccharides in bacteria [41,43]. The presence of multiple WECE-encoding genes and a high expression level of these genes in *K. brevis *suggest that this protein is involved in physiologically important processes in this organism.

#### NAD dependent sugar nucleotide epimerase/dehydratase

**Figure 3:**
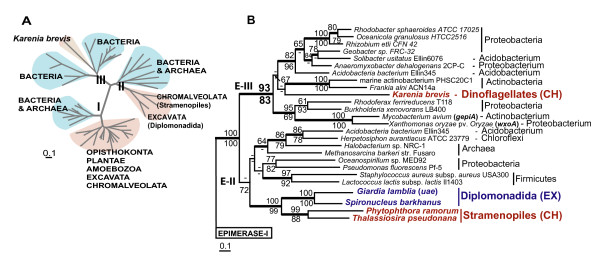
**Phylogeny of NAD-dependent sugar nucleotide epimerase/dehydratase isoforms**. **A**. ML tree of three isoforms of NAD dependent sugar nucleotide epimerase/dehydratase. Light brown color represents eukaryotic clades; blue color represents prokaryotic clades.**B**. A fragment of the ML tree from the Figure 3A representing the phylogeny of NAD dependent sugar nucleotide epimerase/dehydratase isoforms II (E-II) and III (E-III). The numbers above and below the branches are the results of ML and NJ bootstrap analyses, respectively. Only bootstrap values ≥ 60% are shown. The thick branches indicate ≥ 0.95 posterior probability from a Bayesian inference. Branch lengths are proportional to the number of substitutions per site (see the scale bar). CH indicates chromalveolates and EX indicates Excavata. Names of the epimerase-encoding genes that have been studied experimentally [44, 45, 47] are given in brackets.

BLAST searches using sequences of bacterial proteins involved in outer membrane liposaccharide and S-layer glycoprotein biosyntheses [43,44] allowed us to identify two genes encoding NAD dependent sugar nucleotide epimerase in the *K. brevis *EST data. Protein coding regions of the two genes share 98% amino acid sequence identity and significant similarity of their 3'UTR regions. This suggests they arose through a recent gene duplication. Both genes are represented by multiple transcripts and have a GC content typical for *K. brevis *nuclear encoded genes (Table 2). The absence of an N-terminal extension indicates that these sequences likely encode cytosolic proteins. Phylogenetic analyses revealed three related isoforms of NAD dependent sugar nucleotide epimerase that arose through ancient gene duplication in prokaryotes (Fig. 3A). Prokaryotic and major eukaryotic lineages including chromalveolates and Excavata possess epimerase-I. Epimerase-II is present in Bacteria, Archaea, several species of diplomonads (*Giardia lamblia *and *S. barkhanus*), and stramenopiles (*Phytophthora ramorum *and *Thalassiosira pseudonana*; see Additional file 1). Epimerase-III was found only in *K. brevis *and Bacteria. Phylogenetic analyses provide strong support for the monophyly of the *K. brevis *and bacterial epimerase-III sequences (BPml = 93%; BPnj = 83%; BPP = 1.0; Fig. 3B). The tree topology suggests that bacterial epimerase-III was acquired by dinoflagellates through HGT. Epimerase-I, which is present in haptophytes and stramenopiles has not been found in the EST libraries of six dinoflagellate species including *K. brevis*. The distribution and nucleotide composition of the epimerase-III-encoding genes (Table 2) do not allow us to infer the time of this transfer event. The epimerase-II tree strongly supports the monophyly of the two distantly related lineages, diplomonads (Excavata) and stramenopiles (chromalveolates) (BPml = 100%; BPnj = 99%; BPP = 1.0) (Fig. 3B). This tree topology is most easily explained by serial HGT that involved ancient prokaryote-to-eukaryote and more recent eukaryote-to-eukaryote gene transfers (Fig. 2C). Similar to WECE and MVIM, the epimerase tree does not allow us to identify the prokaryotic lineages that contributed these genes to eukaryotes.

The functions of these enzymes have not been studied in chromalveolates. In Bacteria, epimerase-I and III catalyze the biosyntheses of dTDP-D-Fuc *p*3NAc and dTDP-L-ramnose, compounds that serve as precursors for the assembly of outer membrane structural polysaccharides [41-43,45]. On the dTDP-D-Fuc *p*3NAc pathway, they function upstream of the described earlier WECE proteins. We cannot exclude that WECE and epimerase-III represent a functionally coupled enzyme pair in *K. brevis *that might have been acquired by dinoflagellates in one HGT event (Figs. 2, 3). Available experimental data suggest that epimerase-I and II are also involved in cell wall polysaccharide biosynthesis in plants [46] and diplomonads [47]. Based on these data we propose that epimerase-III performs similar function(s) in *K. brevis*.

#### Clavaminic acid synthetase-like protein

Clavaminic acid synthetase (CAS) belongs to the large family of iron and 2-oxoacid-dependent dioxygenases, an important class of enzymes that mediates a variety of oxidative reactions [48]. Most studies of CAS have been carried out using the *Streptomyces *isozymes [49]. In *Streptomyces*, CAS catalyzes three major steps of the clavulanic acid biosynthesis. Clavulanic acid is a natural inhibitor of β-lactamases, enzymes that confer resistance to β-lactam antibiotics in bacteria.

**Figure 4:**
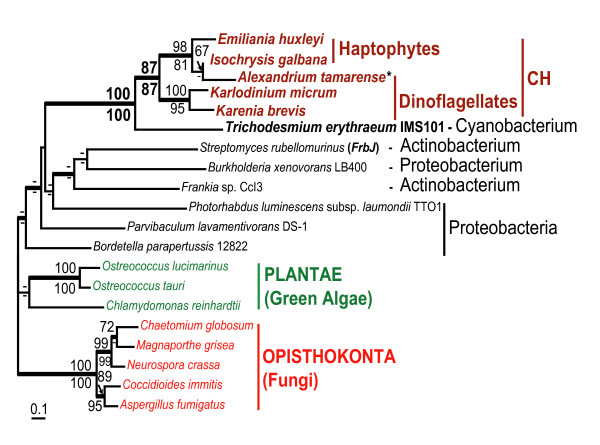
**Origin of clavaminic acid synthetase (CAS) -like protein in Chromalveolata**. ML tree of CAS and CAS-like proteins. The numbers above and below the branches are the results of ML and NJ bootstrap analyses, respectively. Only bootstrap values ≥ 60% are shown. The thick branches indicate ≥ 0.95 posterior probability from Bayesian inference. Branch lengths are proportional to the number of substitutions per site (see the scale bar). CH indicates chromalveolates. The name of the CAS protein-encoding gene that has been studied experimentally [49] is given in brackets. * The position of *Alexandrium tamarense *within the haptophytes clade was inferred from the analysis of a short C-terminal sequence.

We identified homologs of the bacterial CAS proteins in four eukaryotic lineages: Fungi (Opisthokonta), green algae (Plantae), dinoflagellates, and haptophytes (chromalveolates; Fig. 4, see Additional file 1). Partial sequences of three genes encoding CAS-like protein were identified in the *K. brevis *EST data. To the best of our knowledge, the functions of these proteins have not been studied in eukaryotes. According to TargetP and MitoProt, CAS-like proteins in *K. brevis *(P = 0.763 and P = 0.870 respectively), *K. micrum *(P = 0.742 and P = 0.951), and a green alga *Ostreococcus tauri *(P = 0.748 and P = 0.706) are mitochondrial targeted. CAS-like proteins in *O. lucimarinus *and *Chlamydomonas reinhardtii *do not have an N-terminal extension. PSORT [50] predicts a peroxisomal localization for the fungal CAS-like proteins however, with low probability (P-value range = 0.500 – 0.660). Phylogenetic analyses of the CAS-like proteins (Fig. 4) support the monophyly of the two chromalveolate lineages (BPml = 87%; BPnj = 87%; BPP = 0.99) and places chromalveolates in one clade with a cyanobacterium *Trichodesmium erythraeum *IMS101 (BPml = 100%; BPnj = 100%; BPP = 1.0). Most proteins of cyanobacterial origin were derived by chromalveolates from red or green algal progenitors of their plastids via secondary endosymbiosis [25,26]. Absence of Plantae from the cyanobacterial-chromalveolate clade likely indicates that this cyanobacterial protein was acquired by chromalveolates not through endosymbiotic but rather through horizontal gene transfer. Recently, Waller at al. [15] reported another case of cyanobacterium-to-dinoflagellate HGT that involved a DNA fragment encoding the plastid targeted shikimate-O-methyltransferase junction protein. However, presence of the CAS-like proteins in haptophytes suggests a different scenario for the occurrence of this enzyme in dinoflagellates, which might include an additional, haptophyte-to-dinoflagellate HGT. Alternatively the CAS-like protein tree topology could be explained by the gene transfer from cyanobacteria before the divergence of chromalveolates and its subsequent loss from stramenopiles, ciliates, and apicomplexans.

### Energy metabolism

#### Iron-containing alcohol dehydrogenase and NAD-dependent aldehyde dehydrogenase

Iron-containing alcohol dehydrogenase (Fe-ADH) and NAD-dependent aldehyde dehydrogenase (PutA) are probably the best-studied proteins from the perspective of HGT in eukaryotes. Aldehyde-alcohol dehydrogenase protein (AdhE) has arisen through the fusion of two protein domains, PutA and Fe-ADH and is considered to be a key enzyme in energy metabolism in parasitic amitochondriate protists [51]. Previous studies on parasitic protists demonstrated that AdhE-encoding genes have been subjects of multiple independent prokaryote-to-eukaryote HGTs. For information about AdhE functions and phylogeny in parasitic protists we would direct readers to references [51,52]. In addition to previous findings, we identified Fe-ADH in free-living dinoflagellates and jakobids (see Additional file 1). These sequences share over 50% amino acid identity with bacterial homologs. Results of phylogenetic analyses suggest that the two lineages acquired Fe-ADH-encoding genes independently from closely related prokaryotes. The Fe-ADH tree obtained in this study is shown in Additional file 1. PutA has been found in free living dinoflagellates, stramenopiles, and jakobids (see Additional file 1). PutA sequences in these three lineages show over 50% amino acid identity to corresponding bacterial proteins and according to the result of phylogenetic analyses, originated through independent interdomain transfers from distinct prokaryotic donors (see Additional file 1). Phylogenetic analyses strongly support the monophyly of dinoflagellate and bacterial sequences (BPml = 100%; BPnj = 100%; BPP = 1.0) and suggest that dinoflagellates acquired PutA before the divergence of *Karenia *and *Karlodinium*.

#### Malate-quinone oxidoreductase

Malate-quinone oxidoreductase (MQO) is a functional analog of the better-known NAD-dependent malate dehydrogenase (MDH) that catalyses the conversion of malate to oxaloacetate in the tricarboxylic acid (TCA) cycle. In contrast to MDH, bacterial MQO is a membrane-associated enzyme that utilizes flavin adenine dinucleotide (FAD) as a cofactor and donates the electrons from malate oxidation to quinones instead of NAD [53-55]. MQO is protein common in bacteria. Among eukaryotes, this enzyme has been previously reported only for apicomplexans [56]. Apicomplexans lack the mitochondrial form of MDH. It has been shown that MQO compensates for mitochondrial MDH in the TCA cycle in this lineage [57].

**Figure 5:**
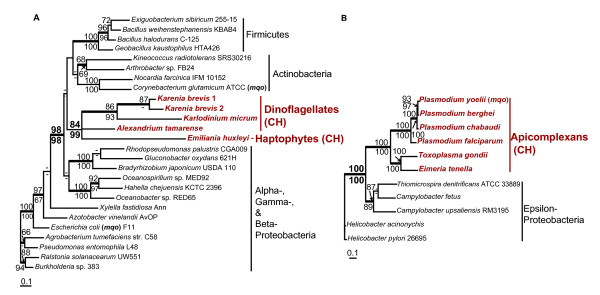
**Origins of two malate/quinone oxidoreductase (MQO) isoforms in Chromalveolata**. **A**. ML tree of MQO isoform DH. B. ML tree of MQO isoform A. The numbers above and below the branches are the results of ML and NJ bootstrap analyses, respectively. Only bootstrap values ≥ 60% are shown. The thick branches indicate ≥ 0.95 posterior probability from a Bayesian inference. Branch lengths are proportional to the number of substitutions per site (see scale bars). CH indicates chromalveolates. Only bacterial sequences that have a BLAST e-value ≤ 10^-20 ^to homologs in eukaryotes are included in these trees. Names of the MQO-encoding genes that have been studied experimentally [54, 55, 57] are given in brackets.

We found two MQO-encoding genes in the *K. brevis *EST data. These share 60% amino acid sequence identity and about 44% identity with their bacterial homologs. Both have nucleotide compositions typical for *K. brevis *nuclear encoded genes (Table 2). Using sequence similarity searches, we identified homologs of the *K. brevis *MQO in several species of dinoflagellates and haptophytes (see Additional file 1). Phylogenetic analyses support the monophyly of the two genes identified in *K. brevis *(BPml = 87%; BPP = 1.0) suggesting that they originated *via *gene duplication after the *Karenia *and *Karlodinium *divergence. Phylogenetic analyses place haptophytes and dinoflagellates within one clade (BPml = 84%; BPnj = 99%; Fig. 5A). The most parsimonious explanation for the observed MQO-DH tree topology is a haptophyte-to-dinoflagellate HGT that occurred early in dinoflagellate evolution.

Surprisingly, comparison of the MQO sequences identified in dinoflagellates and haptophytes (MQO-DH) with the apicomplexan MQO (MQO-A) show that these proteins share significant similarity only at the short N-terminal FAD-binding domains (22% overall amino acid sequence identity). The analysis of the protein distribution and phylogeny showed that MQO-DH and MQO-A have been acquired by chromalveolates from different bacterial donors through independent transfer events (Figs. 5A, 5B). The fact that MQO-A shows highest similarity to homologs in epsilon proteobacteria (all BLAST hits with e-value ≤ 10^-20^) suggests that apicomplexans acquired MQO-A from this bacterial group. Homologs of MQO-DH have been identified in multiple bacterial lineages including firmicutes, actinobacteria, and three proteobacterial groups: alpha-, beta-, and gamma proteobacteria. Although the tree topology (Fig. 5A) does not allow us to identify the bacterial donor of the MQO-DH in chromalveolates, the presence of N-terminal extension in both chromalveolate and proteobacterial MQO-DH sequences suggests a proteobacterial origin of this protein. Highly hydrophobic N-terminal extensions of the proteobacterial MQO sequences are likely responsible for the protein interaction with bacterial membrane. The corresponding regions of the dinoflagellate MQO sequences have a low hydrophobicity and according to the results of analyses with protein topology prediction programs do not encode mitochondrial-, plastid-, peroxisomal-targeting or signal peptides (results not shown). Most likely, MQO-DH represents a cytosolic enzyme. To verify this hypothesis we assessed the presence/absence of MDH isoforms in haptophytes and dinoflagellates. We found a mitochondrial-targeted MDH in both lineages and a cytosolic MDH in haptophytes (see Additional file 1). The cytosolic isoform is absent from EST libraries of six dinoflagellate species that have been analyzed. This observation suggests that cytosolic MDH was replaced by MQO in dinoflagellates, because haptophytes retain both cytosolic enzymes. Analogous cases have been observed in prokaryotes. For example, *Escherichia coli *and *Corynebacterium glutamicum *contain both MQO and MDH [54,55], and *Helicobacter pylori *has only MQO [53]. The study of bacterial MQO shows that reactions catalyzed by this enzyme have a very favorable standard free energy difference (ΔG°) in comparison with reactions catalyzed by MDH [53]. In addition, MQO uses carbon and energy sources different from MDH. Therefore this enzyme may be beneficial for the cell under the conditions unfavorable for MDH activity.

#### Monomeric NADP-dependent isocitrate dehydrogenase

NADP-dependent isocitrate dehydrogenase (NADP-IDH) is an important enzyme of the intermediary metabolism that controls the carbon flux within the TCA cycle and supplies the cell with 2-oxoglutarate and NADPH for biosynthesis [58]. There are several NADP-IDH isoforms in photosynthetic organisms including cytosolic, mitochondrial, plastid, and peroxisomal enzymes. These four NADP-IDH isoforms have arisen in eukaryotes from a single progenitor enzyme [59]. Eukaryotic NADP-IDH proteins form a dimeric structure composed of identical subunits of 40–50 kDa and share about 40% identity to the prokaryotic dimeric NADP-IDH (NADP-IDH-I) [58,59].

**Figure 6:**
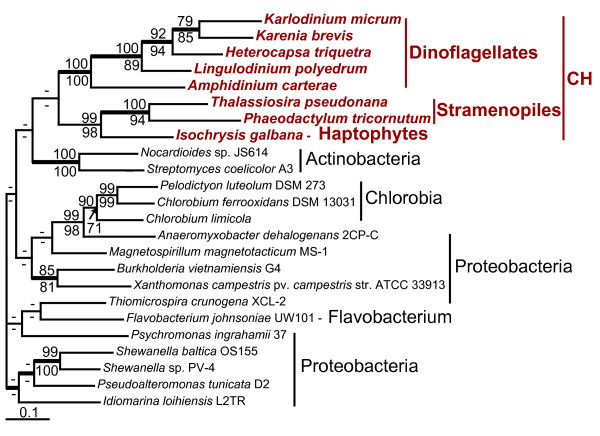
**Origins of NADP-dependent isocitrate dehydrogenase (IDH) in chromalveolates**. ML tree of the chromalveolate-bacterial IDH. The numbers above and below the branches are the results of ML and NJ bootstrap analyses, respectively. Only bootstrap values ≥ 60% are shown. The thick branches indicate ≥ 0.95 posterior probability from a Bayesian inference. Branch lengths are proportional to the number of substitutions per site (see the scale bar). CH indicates chromalveolates.

Analyses of the *K. brevis *EST data allowed us to identify a novel eukaryotic form of NADP-IDH similar to the monomeric NADP-IDH (NADP-IDH-II) found in some bacteria (60% amino acid sequence identity). Apart from prokaryotes, we identified sequences homologous to the *K. brevis *NADP-IDH-II in photosynthetic algae from three chromalveolate lineages: dinoflagellates, stramenopiles, and haptophytes (Fig. 6; see Additional file 1). The structure and distribution of chromalveolate NADP-IDH-II-encoding genes suggest this protein is plastid targeted. These proteins contain plastid targeting N-terminal extensions composed of a 22–23 aa signal peptide (P > 0.9) and a 48–65 aa plastid targeting peptide that are typical for chromalveolates [60,61]. The inventory of IDH isoforms in chromalveolates showed that plastid NADP-IDH-I is absent from this lineage. Presumably, NADP-IDH-II was acquired by the chromalveolate ancestor from an unidentified bacterial donor at the time of plastid establishment and consequently lost from several chromalveolate lineages including ciliates, apicomplexans, and non-photosynthetic stramenopiles. The gene losses correlate with the loss of photosynthetic ability in these lineages, which suggests the involvement of the enzyme in photosynthesis-related processes. In bacteria, NADP-IDH-II performs the same functions as NADP-IDH-I. Most extant bacteria have only one of these enzymes. Experimental study of IDH activity in the marine bacterium, *Colwellia maris*, which uniquely possesses both IDHs, showed that NADP-IDH-II contributes a molecular basis for cold adaptation in this species [62]. NADP-IDH-II demonstrated maximum biochemical activity at 4°C and was completely inactivated above 20°C. Furthermore, this enzyme has been shown to enable *E*. *coli *mutants to grow at low temperature.

#### Substrate-bound periplasmic binding protein

Bacterial substrate-bound periplasmic binding proteins (PBPb) are components of membrane-associated complexes that transport a wide variety of substrates, such as, amino acids, peptides, sugars, vitamins, and inorganic ions [63]. We found homologs of a bacterial PBPb in three lineages of photosynthetic chromalveolates and a photosynthetic excavate *Euglena gracilis *(see Additional file 1). Although PBPb has a restricted distribution similar to NADP-IDH-II in photosynthetic eukaryotes, analyses with protein topology prediction programs do not support a plastid localization of PBPb. Phylogenetic analyses support the monophyly of chromalveolate and *E. gracilis *PBPb sequences (see Additional file 1) suggesting that, like MVIM-WECE and epimerase-II, this protein spread among eukaryotes through intradomain HGT.

#### Translation elongation factor 2

**Figure 7:**
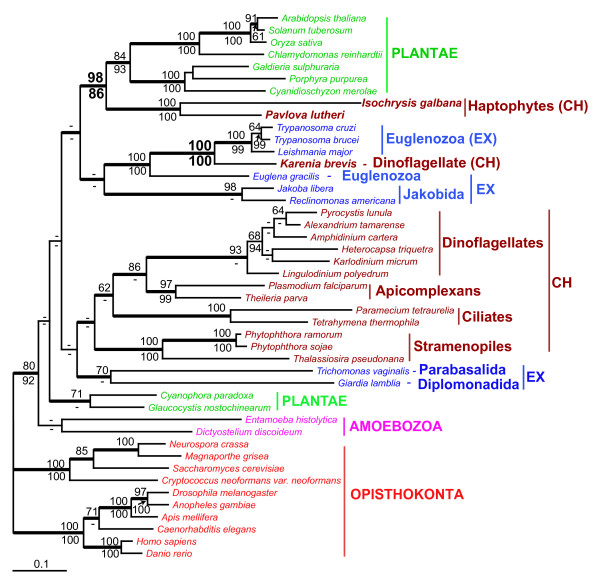
**Phylogeny of translation elongation factor 2 (EF2) in eukaryotes**. ML tree of EF2. The numbers above and below the branches are the results of ML and NJ bootstrap analyses, respectively. Only bootstrap values ≥ 60% are shown. The thick branches indicate ≥ 0.95 posterior probability from a Bayesian inference. Branch lengths are proportional to the number of substitutions per site (see the scale bar). CH indicates chromalveolates and EX indicates Excavata.

Translation elongation factor 2 (EF2) is a Ca^2+^/calmodulin-dependent protein kinase III involved in protein synthesis in eukaryotes [64]. EF2 is responsible for the translocation of the peptidyl-tRNA from the acceptor site to the peptidyl-tRNA site of the ribosome, thereby freeing the A-site for the binding of the next aminoacyl-tRNA. Highly conserved among eukaryotes, EF2 is considered to be a robust phylogenetic marker. This protein has been successfully used for phylogenetic analyses of Plantae and Opistokonta [65-67]. However, our attempt at using EF2 for inferring protist phylogeny demonstrated that the gene encoding this protein has been subject to HGT in several chromalveolate lineages (Fig. 7). Our phylogenetic analysis provides strong support (BPml = 100%; BPnj = 100%; BPP = 1.0) for the position of the *K. brevis *EF2 within Euglenozoa (Excavata). The chromalveolate type of EF2 identified in six species of dinoflagellates including *K. micrum *is absent from the *K. brevis *EST data. The tree topology and protein distribution suggest that the euglenozoa-derived EF2 replaced the chromalveolate homolog of this protein after the *Karenia *and *Karlodinium *divergence. Analyses of the nucleotide composition of EF2-encoding genes support the phylogenetic inferences (Table 2). The GC content of the *K. brevis *EF2 gene is significantly higher than for typical nuclear genes in this taxon. The nucleotide composition of EF2-encoding genes in *K. micrum *and other chromalveolates does not deviate from the GC content range identified for these species. This observation suggests that EF2 was recently acquired by *Karenia *from a GC-rich excavate donor. Haptophyte algae represent another chromalveolate lineage that possesses a non-chromalveolate form of this protein. Under chromalveolate monophyly [68], the position of haptophytes as a sister group of Plantae (BPml = 98%; BPnj = 86%; BPP = 1.0) in the EF2 tree should be interpreted as evidence that this protein was acquired by haptophytes through HGT (Fig. 7). However, due to the fact that the phylogenetic position of the haptophyte-cryptophyte clade relative to other chromalveolate lineages remains unresolved [21,22], this leaves uncertain the origin of this protein in haptophytes.

Phylogenetic trees and sequence information for the remaining seven proteins putatively acquired by chromalveolates via HGT (see Table 1) are provided in Additional file 1.

## Discussion

### Recurrent HGT in protists

The results of our study demonstrate that the recurrent non-genealogical influx of genetic material from various prokaryotic and eukaryotic donors is an important contributor to genome evolution in chromalveolates. Although our data screening approach was aimed at detecting only chromalveolate-specific HGTs, detailed analyses of the identified proteins revealed that many of them are present in several eukaryotic lineages. A co-occurrence of bacteria-derived genes encoding the same enzyme in a limited number of species from distantly related eukaryotic lineages has been reported previously [8,13-15,51,69-72] (Table 3).

**Table 3:** Gene distribution by multiple independent HGTs in eukaryotes

**Protein**	**Multiple Independent HGTs**		**Reference**
	**mode**	**eukaryotic lineages involved**	
Fructose-bisphosphate aldolase class II, type B	p-eu, eu-eu	Excavata, Amoebozoa	[69]
Pyruvate phosphate dikinase	p-eu	Excavata	[69]
Translation elongation factor-1 alpha-like protein	eu-eu	Excavata, Chromalveolata, Opisthokonta, Plantae	[13]
Shikimate biosynthetic enzyme AroB	p-eu	Chromalveolata, Opisthokonta (Fungi)	[15]
Hybrid-cluster protein	p-eu, eu-eu	Excavata, Chromalveolata, Amoebozoa, Plantae (Green algae)	[51]
A-type flavoprotein	p-eu, eu-eu	Excavata, Amoebozoa	[51]
Glucosamine-6-phosphate isomerase	p-eu, eu-eu	Excavata, Chromalveolata, Amoebozoa, Opisthokonta (Fungi)	[51]
Alcohol dehydrogenase	p-eu, eu-eu	Excavata, Chromalveolata, Amoebozoa, Plantae (Green algae), Opisthokonta (Fungi)	[51]
Glutamate dehydrogenase	p-eu	Excavata, Chromalveolata	[70]
Glyceraldehyde-3-phosphate	p-eu, eu-eu	Excavata, Chromalveolata	[14]
Alanyl-tRNA synthetase	p-eu, eu-eu	Excavata, Chromalveolata, Amoebozoa	[71]
Arginine deiminase	p-eu, eu-eu	Excavata, Amoebozoa	[8]
Rubrerythrin	p-eu	Excavata, Amoebozoa	[8]
Hypothetical protein	p-eu, eu-eu	Excavata, Amoebozoa	[72]

The "p-eu" indicates interdomain prokaryote-to-eukaryote HGT. The "eu-eu" indicates intradomain eukaryote-to-eukaryote HGT.

Two features typical for phylogenetic trees resulting from the analysis of these proteins are: (1) the presence of several prokaryotic-eukaryotic clades within one tree (Fig. 3, Additional file 1) and (2) the presence of several species from distantly related eukaryotic lineages within one clade (Figs. 2, 3, 4, 5, 7, Additional file 1). These tree topologies may be explained by multiple independent inter- and intradomain transfers of genes encoding the same enzyme. The study of HGT in bacteria and parasitic protists demonstrates that adaptation to specific environments is the major force driving HGT [33,72]. Genes beneficial under certain environmental conditions can independently be acquired by different eukaryotic lineages that occupy different niches. Reconstructing the phylogeny of proteins involved in anaerobic glycolysis in parasitic protists provides an illustration of this scenario [69]. Here, the gene encoding fructose-bisphosphate aldolase class II, type B was acquired independently by Parabasalida and the common ancestor of Oxymonadida and Diplomonadida. Three prokaryote-to-eukaryote transfers explain the occurrence of pyruvate phosphate dikinase in Parabasalida, parasitic Euglenozoa, and Oxymonadida-Diplomonadida lineage. The aerobe-to-anaerobe transition occurred several times in the evolution of excavates. During this transition, different lineages of excavates independently acquired genes associated with anaerobic glycolysis from prokaryotes that had already inhabited corresponding niches.

The results of our study show that this scenario is applicable as well to free-living eukaryotes. Two isoforms of sugar epimerase, epimerase-II and epimerase-III that originated via an ancient gene duplication event in prokaryotes were independently acquired by dinoflagellates and stramenopiles (Fig. 3). Two independent interdomain HGTs explain the occurrence of structurally distinct isoforms of bacterial MQO in free living haptophytes and dinoflagellates and parasitic apicomplexans. The second feature of phylogenetic trees resulted from the analysis of transferred genes, the monophyly of distantly related eukaryotes, may be explained either by intradomain (eukaryote-to-eukaryote) gene transfer or by several interdomain transfers from the same prokaryotic donor. This type of phylogeny is more likely to reflect specific relationships between microorganisms that occupy (or occupied in their evolutionary past) one ecological niche. Sequential HGTs that involved a prokaryote and two distantly related anaerobic protists have been previously proposed as an explanation for the patchy distribution of alcohol dehydrogenase, alanyl-tRNA synthetase, and fructose-bisphosphate aldolase class II, type B protein among eukaryotes [51,69,71] (Table 3). The bacteria-derived isoform of glyceraldehyde-3-phosphate that functions as a cytosolic protein in free living dinoflagellates and *Euglena *and as a glycosomal protein in parasitic Euglenozoa provides another example of a protein derived by eukaryotes through sequencial HGTs [14] (Table 3). Gene acquisition through sequential HGTs is the most plausible scenario for the distribution of MVIM-WECE, epimerase-II, PBPb, and, possibly, MQO-DH and CAS-like protein presented in this paper. It is believed that HGT is more likely to occur between closely related lineages [73]. Such transfers are hard to identify unless transferred genes have a limited distribution within the studied taxonomic group. CAS-like protein and MQO-CH identified in this study are present in two chromalveolate lineages, haptophytes and dinoflagellates (Figs. 4, 5). Possible interpretations of a patchy gene distribution between closely related lineages include differential gene loss and gene transfer. The gene loss scenario would assume an independent gene loss from three chromalveolate lineages: stramenopiles, ciliates, and apicomplexans. However, the fact that haptophytes provide not only a food source but also a unique pool of temporary plastids (kleptoplastids) for several species of extant dinoflagellates [74,75] and have contributed the plastid to the common ancestor of *Karenia *and *Karlodinium *[32,76] demonstrates that these two relatively distantly related algal lineages have been involved in specific predator-prey interactions over millions of years. This fact makes sequential HGTs a more plausible scenario for the occurrence of MQO-CH and CAS-like protein in dinoflagellates.

Genes encoding MVIM, WECE, epimerase-II, and PBPb proteins are shared by bacteria and several lineages of chromalveolates and excavates. According to the results of our phylogenetic analyses, genes encoding these proteins were acquired by one eukaryotic lineage through an ancient interdomain HGT and transferred to another *via *intradomain HGT. Phagotrophy is widespread in chromalveolates and excavates therefore this feeding mode may explain an increased rate of HGT in these taxa [11,52,72,77]; i.e., many extant species of excavates and chromalveolates feed on bacterial and eukaryotic microorganisms [78-82]. This dynamic process has made it impossible to identify donors and recipients in these eukaryote-to-eukaryote HGTs. An inconsistency between the gene phylogeny and species phylogeny observed in the prokaryotic region of the MVIM, WECE, and epimerase trees suggests that genes encoding these proteins are subjects of frequent HGTs in bacteria. Structural analyses of bacterial gene clusters that include close homologs of the *K. brevis *WECE (*ftdB *and *qdtB*) and epimerase-III (*gepiA *and *wxoA*) support this scenario [41,45]. *FtdB *and *qdtB *belong to the large cluster of genes involved in the biosynthesis of surface layer glycoproteins (SLG) in firmicutes. It has been shown that the GC content of the SLG clusters deviates significantly in many bacteria from the GC content of genome as a whole [41]. This observation together with the fact that SLG clusters are typically flanked by several transposases or remnants thereof indicate that the entire SLG region may be a subject of HGT in bacteria. A similar conclusion resulted from the analysis of the bacterial lipopolysaccharide biosynthetic loci that includes *gepiA *and *wxoA *[45]. This observation completes the proposed scenario of gene distribution by sequential HGTs with an additional feature that is prokaryote-to-prokaryote HGT.

### Measuring the contribution of HGT to eukaryotic genomes

Several attempts to numerically estimate the contribution of HGT to eukaryotic genomes suggest a substantial inter-taxon variation in the number of horizontally derived genes [7,12,52,83,84]. Existing studies show that although extremely rare in Plantae and multicellular Opisthokonta, HGT is a common phenomenon in Amoebozoa, Excavata, and chromalveolates. However, the variation in the numbers of HGTs reported for different species within the phagotrophic lineages (see Background) reflects a difference in analytical approaches. Differences in stringency of data screening parameters, the taxonomic composition of databases used for the comparative analysis, and methods of phylogenetic analyses can significantly affect the outcome of the study. Standardization of methods for estimating the contribution of HGT in eukaryotic genomes should be based on the knowledge of tempo and mode of evolution of horizontally transferred genes. To our knowledge, these issues have never been exhaustively studied in eukaryotes.

Studies of prokaryotic genome evolution demonstrate that many recently transferred genes have very large K_A_/K_S _ratio that suggests directional selection [73]. In addition, the rate of duplications among genes derived through HGTs is significantly higher than among indigenous ones in bacteria [85]. The proposed scenario for the fate of transferred genes in bacteria based on these observations includes their uptake, duplication, rapid diversification of gene copies by mutations, and consequent fixation of the "best" copies and elimination of other duplicates. Is this scenario applicable for eukaryotes?

Analyses of proteins presented in these study show that nine of them are encoded by at least 2–12 genes in the *K. brevis *genome (Table 1). Following the standard approach of phylogenomics, we excluded from the analysis all proteins represented by multiple paralogs in several eukaryotic lineages. Therefore we investigated only those paralogs that arose from relatively recent duplications. The fact that several dinoflagellate species contain multiple highly divergent (< 50% amino acid identity) paralogs of ATS1 (Additional file 1) suggests that duplication of genes encoding this protein occurred before the divergence of dinoflagellate lineages. Phylogenetic analyses of ATS1 support this statement (see Additional file 1). The amino acid sequence identity of paralogs resulting from duplications that, according to phylogenetic analyses, occurred after the *Karenia *and *Karlodinium *divergence varies from 60% (MQO) to 99% (WECE). The comparison of genes encoding WECE protein shows that the amino acid sequence identity of different copies varies from 81 to 99%. Assuming that divergence between two paralogs is proportional to the time elapsed since gene duplication, the observed variation suggests that duplication of WECE genes is a continuous process in *K. brevis*.

To summarize, the results of this analysis demonstrate that the impact of HGT on genome evolution in dinoflagellates is reinforced by continuous duplications of the transferred genes and consequent diversification of the resulting paralogs. Additional studies are required to estimate relative duplication rates of foreign and indigenous genes, rates of mutations, and paralog silencing in this group of organisms.

## Conclusion

Taking into consideration that genomic data are available for only a minuscule fraction of bacteria and protists populating our planet and that we were able to identify multiple cases of HGT of genes encoding the same proteins leads us to one simple conclusion. Horizontal gene transfer contributes significantly to protist genomes. We believe that in niches where parasitism and phagotrophy are common, beneficial genes may spread rapidly from prokaryotes to eukaryotes and provide a molecular basis for niche-specific adaptations in the latter group. It is clear however, that all genes are not transferred with equal frequency in eukaryotes with the majority of HGT candidates being involved in metabolic processes. However, given that foreign DNA fragments from eukaryotes frequently integrate in protist chromosomes, it should not be surprising that occasionally genes encoding proteins of a more universally conserved function such as EF2 and potentially EF-1 alpha-like [13] may also be co-transferred. Apart from the exciting ramifications for post-HGT gene evolution in eukaryotes that includes gene family evolution and selection for novel functions, our work also underlines the great care that needs to be taken when generating eukaryote-wide trees of life that include many phagotrophic or parasitic taxa.

## Methods

### *Karenia brevis *EST library

In this study, we used EST data generated from clonal *K brevis *Wilson cells grown under five different culture conditions: 1) under nitrate depletion, 2) under phosphate depletion, 3) in log phase under replete conditions, harvested during the light phase, 4) in the presence of oxidative metals, and 5) undergoing heat stress. For complete information about generation, sequencing, and processing of the *K. brevis *EST library see reference [25]. Clustering and assembly of the EST was done using default settings of the TGICL computer program [86,87]. The assembly resulted in 9,786 EST contigs; each representing a unique gene.

### Identification of proteins acquired by chromalveolates through ancient HGTs

To identify ancient HGTs in chromalveolates, we analyzed a subset of genes present in the *K. brevis *EST data and in the EST data of at least one other species of dinoflagellate. This approach allowed us to exclude from the analysis possible bacterial contaminants of the EST library. Genes shared by dinoflagellates have been detected using *K. brevis *ESTs as an input for the sequence similarity search (BLAST; e-value ≤ 10^-10^) against a local database that included available data from the GenBank dbEST database [18] for five dinoflagellate species: *Alexandrium tamarense*, *Amphidinium carterae*, *Heterocapsa triquetra*, *Lingulodinium polyedrum*, and *K. micrum*. This analysis yielded 3,341 EST contigs. To detect potential HGTs, we used the defined subset of *K. brevis *DNA sequences as an input for the sequence similarity search (BLASTx; e-value ≤ 10^-20^) against the GenBank non-redundant database (nr). Sequences that showed highest similarity to prokaryotic proteins (three top hits) or chromalveolate and prokaryotic proteins have been selected for further analyses. Using this approach, we identified 95 *K. brevis *unigenes encoding proteins from 55 protein families putatively derived from prokaryotes at different time points of chromalveolate evolution.

In parallel, we performed a high throughput automated analysis of the subset of sequences shared by dinoflagellates. The 3,341 sequences were translated into the six open reading frames using the Transeq program in the Emboss package [88] and used as input for the analysis with the PhyloGenie package of computer programs [89]. PhyloGenie serves as an automated pipeline in which the following analyses can be implemented: BLAST search against a local database, extraction of homologous sequences from the BLAST results, generation of alignments, phylogenetic tree reconstruction, and calculation of bootstrap support values for individual phylogenies. We created a local protein database for the PhyloGenie BLAST search by retrieving completed genome sequences and EST data from the National Center for Biotechnology Information (NCBI) [90] genomic projects web site and dbEST [18], DOE Joint Genome Institute (JGI) [91], *Cyanidioschyzon merolae *genome project [92], and The *Galdieria sulphuraria *genome project [93] for species listed below. EST sequences have been translated into six open reading frames and combined with protein sequences. The final fasta file that included all of the data was formatted using the formatdb program in the BLAST package [94]. Our local database included complete genome and EST data for following species: *Oryza sativa*, *Drosophila melanogaster*, *Saccharomyces cerevisiae*, the green alga *Chlamydomonas reinhardtii*, red algae *C. merolae *and *G. sulphuraria*; chromalveolates *Guillardia theta*, *Emiliania huxleyi*, *Thalassiosira pseudonana*, *Plasmodium falciparum*, *Toxoplasma gondii*, *A. tamarense*, *A. carterae*, *H. triquetra*, *L. polyedrum*, and *K. micrum*, excavates *G. lamblia *and *Trypanosoma brucei*, archaea *Halobacterium *sp. NRC-1, *Methanothermobacter thermautotrophicus*, and *Sulfolobus tokodaii*, and eubacteria *Clostridium acetobutylicum *ATCC824, *Escherichia coli *536, *Geobacter sulfurreducens*, *Oceanobacillus iheyensis*, *Synechococcus elongatus *PCC 7942, *Trichodesmium erythraeum*, and *Nostoc *sp. PCC 7120.

PhyloGenie was run using default settings except that the minimum expect "e-value" for the BLAST search of the data was set at 10. The hidden Markov model (hmm) alignments were built using all hits with an e-value below 0.01. The program TreeView [95] was used to visualize the resulting trees. We selected sequences represented by trees that contained only chromalveolates and bacteria and trees that contained well-defined chromalveolate-bacterial clades (at least 50% bootstrap support). Using this criterion for the gene selection, we excluded from the analyses genes of eukaryotic and mitochondrial origin that are shared by most eukaryotic organisms. In addition, this approach allowed us to exclude from the analyses genes of red algal and green algal origin acquired by chromalveolates from the genomes of plastid progenitors *via *EGT (see [25,26] for detailed analyses of EGT in chromalveolates). This analysis yielded 37 unigenes encoding proteins from 23 different protein families; 22 of them represented a subset of proteins identified using the "Best hit" approach. The necessity of using a combination of two described above methods for detecting putative HGTs resulted from the fact that neither the non-redundant (nr) nor our local database included all taxa of interest. The local database for the PhyloGenie BLAST search complemented nr with complete genome and EST data for free living protists. In addition to the gene discovery, the PhyloGenie output was used to verify the phylogeny of proteins identified using the "Best hit" approach. Based on the results of PhyloGenie, eight proteins represented by 12 unigenes have been excluded from the analysis as derived from the genome of plastid progenitor through EGT. Three proteins represented by five unigenes were rejected as shared by multiple eukaryotic lineages. The remaining set of 45 proteins represented by 80 unigenes in the *K. brevis *EST data were subjects for detailed analyses.

### Identification of bacteria-derived proteins in *K. brevis *involved in cell wall biogenesis

In bacteria, genes encoding physiologically coupled reactions are often transferred together, frequently in an operon [33]. To test whether this scenario is applicable for interdomain prokaryote-to-eukaryote transfers, we screened the *K. brevis *EST data for the presence of homologs of bacterial proteins involved in cell envelope biogenesis. This category of proteins was chosen to identify genes that potentially may be co-transferred with genes encoding MVIM-WECE. The latter represents a rare case of gene fusion in interdomain HGT. Gene clusters involved in the surface layer protein biosynthesis in *A. thermoaerophilus *[43] and glycopeptidolipid biosynthesis in *Mycobacterium avium *[44] were retrieved from GenBank and used as an input in sequence similarity search (BLAST; e-value ≤ 10^-20^) against the non-redundant gene set generated from the *K. brevis *EST data. This analysis allowed us to identify three additional proteins represented by five unigenes in the *K. brevis *EST data.

### Identification of eukaryotic proteins acquired by chromalveolates through intradomain HGT

Genes derived through intradomain eukaryote-to-eukaryote HGT were extremely hard to identify using high throughput phylogenomic analyses due to the limited number of taxa included in our local database. The only candidate for the transfer of a bona fide eukaryotic gene among taxa is EF2 that was identified in the course of another study of potential markers for reconstructing the eukaryotic tree of life. The detailed description of methods used for the EF2 analysis can be found in reference [21].

To assess the possibility that the EF2 sequence found in the *K. brevis *data resulted from contamination of the EST library with kinetoplastid DNA, we used the complete *K. brevis *EST data set as an input for a sequence similarity search (BLASTx; e-value ≤ 10^-10^) against the GenBank non-redundant database (nr). Sequences that showed the highest similarity to kinetoplastid genes were subjected to detailed analyses. This work did not provide any support for the kinetoplastid origin of identified sequences in *K. brevis *with the exception of EF2 (results not shown).

### Building the final alignments

To build the final alignments, we identified homologs of the candidate *K. brevis *sequences using BLAST searches (e-value ≤ 10^-10^) against GenBank nr, dbEST, and public protist databases including the JGI database [91], the French National Sequencing Center, Genoscope [96], the Protist EST Program database [17], *C. merolae *[92,97] and *G. sulphuraria *[93,98] databases. The DNA sequences were translated, and the amino acid data for each protein were manually aligned with the identified bacterial and eukaryotic homologs using BioEdit [99]. Only regions that were unambiguously aligned were retained for phylogenetic analysis.

### Analysis of sequence structure and phylogeny

The eukaryotic structure of the identified *K. brevis *transcripts has been verified by translating and aligning the resulting amino acid sequences with their bacterial homologous. The subcellular localization of the studied proteins was predicted using online analyses with protein topology prediction programs SignalP [100,101], TargetP [102,103], MITOPROT [104], PSORT [50], and TMHMM [31]. Percent of amino acid sequence identity between gene copies was inferred using an online tool for pair wise sequence alignment bl2seq [105]. GC content of nucleotide sequences was identified using BioEdit. The average and range of nucleotide composition of protist genomes was inferred from the analysis of coding regions of 50 sequences from each species.

We used the maximum likelihood (ML) method to reconstruct the gene phylogenies. The ML analysis was done in PHYML V2.4.3 [106,107] using the WAG + Γ + I evolutionary model and tree optimization. The alpha values for the gamma distribution were calculated using eight rate categories. To test the stability of monophyletic groups in the ML trees, we calculated PHYML bootstrap (100 replicates) support values [108]. In addition, we calculated bootstrap values (500 replications) using the neighbor joining (NJ) method with JTT+Γ distance matrices using PHYLIP V3.63 [109]. The NJ analysis was done with randomized taxon addition. Bayesian posterior probabilities for nodes in the ML tree were calculated using MrBayes V3.0b4 [110] and the WAG + Γ model. The Metropolis-coupled Markov chain Monte Carlo from a random starting tree was run for 1,000,000 generations with trees sampled each 1,000 cycles. The initial 20,000 cycles (200 trees) were discarded as the "burn in." A consensus tree was made with the remaining 800 phylogenies to determine the posterior probabilities at the different nodes.

The names of the *K. brevis *proteins are derived from the names of corresponding protein domains according to the Pfam [29,30] nomenclature. The *K. brevis *sequences listed in the Table 1 have been deposited in GenBank under accession numbers EF540322–EF540340.

## Competing interests

The author(s) declare that there are no competing interests.

## Authors' contributions

TN did the phylogenetic and other bioinformatic analyses and prepared the manuscript draft. DB conceived of and supervised this study and edited the manuscript. All authors read and approved the final manuscript.

## Supplementary Material

Additional file 1 Supporting materials for the study. The complete list of sequences used in this study is shown as well as three figures that show the origins of iron-containing alcohol dehydrogenase (Fe-ADH) and NAD-dependent aldehyde dehydrogenase (PutA) in eukaryotes by multiple HGTs, the origins of substrate-bound periplasmic binding protein (PBPb) and silent information regulator 2 (SIR2) in protists, and examples of HGT events from bacteria to dinoflagellates. Click here for file

## References

[B1] Ochman H, Lawrence JG, Groisman EA (2000). Lateral gene transfer and the nature of bacterial innovation. Nature.

[B2] Boucher Y, Douady CJ, Papke RT, Walsh DA, Boudreau ME, Nesbo CL, Case RJ, Doolittle WF (2003). Lateral gene transfer and the origins of prokaryotic groups. Annu Rev Genet.

[B3] Dagan T, Martin W (2007). Ancestral genome sizes specify the minimum rate of lateral gene transfer during prokaryote evolution. Proc Natl Acad Sci USA.

[B4] Goldenfeld N, Woese C (2007). Biology's next revolution. Nature.

[B5] Prince VE, Pickett FB (2002). Splitting pairs: the diverging fates of duplicated genes. Nat Rev Genet.

[B6] Ohno S (1970). Evolution by Gene Duplication.

[B7] Loftus B, Anderson I, Davies R, Alsmark UC, Samuelson J, Amedeo P, Roncaglia P, Berriman M, Hirt RP, Mann BJ (2005). The genome of the protist parasite *Entamoeba histolytica*. Nature.

[B8] Andersson JO, Sjogren AM, Horner DS, Murphy CA, Dyal PL, Svard SG, Logsdon JM, Ragan MA, Hirt RP, Roger AJ (2007). A genomicsurvey of the fish parasite *Spironucleus salmonicida *indicates genomic plasticity among diplomonads and significant lateral gene transfer in eukaryote genome evolution. BMC Genomics.

[B9] Carlton JM, Hirt RP, Silva JC, Delcher AL, Schatz M, Zhao Q, Wortman JR, Bidwell SL, Alsmark UC, Besteiro S (2007). Draft genome sequence of the sexually transmitted pathogen *Trichomonas vaginalis*. Science.

[B10] Huang J, Mullapudi N, Lancto CA, Scott M, Abrahamsen MS, Kissinger JC (2004). Phylogenomic evidence supports past endosymbiosis, intracellular and horizontal gene transfer in *Cryptosporidium parvum*. Genome Biol.

[B11] Ricard G, McEwan NR, Dutilh BE, Jouany JP, Macheboeuf D, Mitsumori M, McIntosh FM, Michalowski T, Nagamine T, Nelson N (2006). Horizontal gene transfer from bacteria to rumen ciliates indicates adaptation to their anaerobic, carbohydrates-rich environment. BMC Genomics.

[B12] Eichinger L, Pachebat JA, Glockner G, Rajandream MA, Sucgang R, Berriman M, Song J, Olsen R, Szafranski K, Xu Q (2005). The genome of the social amoeba *Dictyostelium discoideum*. Nature.

[B13] Keeling PJ, Inagaki Y (2004). A class of eukaryotic GTPase with a punctate distribution suggesting multiple functional replacements of translation elongation factor 1alpha. Proc Natl Acad Sci USA.

[B14] Takishita K, Ishida K, Maruyama T (2003). An enigmatic GAPDH gene in the symbiotic dinoflagellate genus *Symbiodinium *and its related species (the order Suessiales): possible lateral gene transfer between two eukaryotic algae, dinoflagellate and euglenophyte. Protist.

[B15] Waller RF, Slamovits CH, Keeling PJ (2006). Lateral gene transfer of a multigene region from cyanobacteria to dinoflagellates resulting in a novel plastid-targeted fusion protein. Mol Biol Evol.

[B16] Archibald JM, Rogers MB, Toop M, Ishida K, Keeling PJ (2003). Lateral gene transfer and the evolution of plastid-targeted proteins in the secondary plastid-containing alga *Bigelowiella natans*. Proc Natl Acad Sci USA.

[B17] The Protist EST Program database (TBestDB). http://megasun.bch.umontreal.ca/pepdb/pepdb.html.

[B18] NCBI. Expressed Sequence Tags database. http://www.ncbi.nlm.nih.gov/dbEST/index.html.

[B19] Cavalier-Smith T (1999). Principles of protein and lipid targeting in secondary symbiogenesis: euglenoid, dinoflagellate, and sporozoan plastid originsand the eukaryote family tree. J Eukaryot Microbiol.

[B20] Parfrey L, Barbero E, Lasser E, Dunthorn M, Bhattacharya D, Patterson D, Katz L (2006). Evaluating support for the current classification of eukaryotic diversity. PLoS.

[B21] Hackett JD, Yoon HS, Li S, Reyes-Prieto A, Rummele SE, Bhattacharya D (2007). Phylogenomic analysis supports the monophyly of cryptophytes and haptophytes and the association of 'Rhizaria' with chromalveolates. Mol Biol Evol.

[B22] Patron NJ, Inagaki Y, Keeling PJ (2007). Multiple gene phylogenies support the monophyly of cryptomonad and haptophyte host lineages. Curr Biol.

[B23] Archibald JM, Keeling PJ (2002). Recycled plastids: a 'green movement' in eukaryotic evolution. Trends Genet.

[B24] Hackett JD, Yoon HS, Soares MB, Bonaldo MF, Casavant TL, Scheetz TE, Nosenko T, Bhattacharya D (2004). Migration of the plastid genome to the nucleus in a peridinin dinoflagellate. Curr Biol.

[B25] Nosenko T, Lidie KL, Van Dolah FM, Lindquist E, Cheng JF, Bhattacharya D (2006). Chimeric plastid proteome in the Florida "red tide"dinoflagellate *Karenia brevis*. Mol Biol Evol.

[B26] Li S, Nosenko T, Hackett JD, Bhattacharya D (2006). Phylogenomic analysis identifies red algal genes of endosymbiotic origin in the chromalveolates. Mol Biol Evol.

[B27] Patron NJ, Waller RF, Keeling PJ (2006). A tertiary plastid uses genes from two endosymbionts. J Mol Biol.

[B28] Fleming LE, Backer LC, Baden DG (2005). Overview of aerosolized Florida red tide toxins: exposures and effects. Environ Health Perspect.

[B29] Sonnhammer EL, Eddy SR, Durbin R (1997). Pfam: a comprehensive database of protein domain families based on seed alignments. Proteins.

[B30] Bateman A, Birney E, Cerruti L, Durbin R, Etwiller L, Eddy SR, Griffiths-Jones S, Howe KL, Marshall M, Sonnhammer EL (2002). The Pfam protein families database. Nucleic Acids Res.

[B31] TMHMM Server v. 2.0. Prediction of transmembrane helices in proteins. http://www.cbs.dtu.dk/services/TMHMM-2.0/.

[B32] Yoon HS, Hackett JD, Van Dolah FM, Nosenko T, Lidie KL, Bhattacharya D (2005). Tertiary endosymbiosis driven genome evolution in dinoflagellate algae. Mol Biol Evol.

[B33] Pal C, Papp B, Lercher MJ (2005). Adaptive evolution of bacterial metabolic networks by horizontal gene transfer. Nat Genet.

[B34] Xue Y, Zhao L, Liu HW, Sherman DH (1998). A gene cluster for macrolide antibiotic biosynthesis in *Streptomyces venezuelae*: architecture of metabolic diversity. Proc Natl Acad Sci USA.

[B35] Xue Y, Wilson D, Zhao L, Liu H, Sherman DH (1998). Hydroxylation of macrolactones YC-17 and narbomycin is mediated by the *pikC*-encoded cytochrome P450 in *Streptomyces venezuelae*. Chem Biol.

[B36] Anzai Y, Saito N, Tanaka M, Kinoshita K, Koyama Y, Kato F (2003). Organization of the biosynthetic gene cluster for the polyketide macrolide mycinamicin in *Micromonospora griseorubida*. FEMS Microbiol Lett.

[B37] Brikun IA, Reeves AR, Cernota WH, Luu MB, Weber JM (2004). The erythromycin biosynthetic gene cluster of *Aeromicrobium erythreum*. J Ind Microbiol Biotechnol.

[B38] Awram P, Smit J (2001). Identification of lipopolysaccharide O antigen synthesis genes required for attachment of the S-layer of *Caulobacter crescentus*. Microbiology.

[B39] Bastin DA, Reeves PR (1995). Sequence and analysis of the Oantigen gene (rfb) cluster of *Escherichia coli *O111. Gene.

[B40] Nesper J, Kraiß A, Schild S, Blaß J, Klose KE, Bockemühl J, Reidl J (2002). Role of *Vibrio cholerae *O139 surface polysaccharides in intestinal colonization. Infect Immun.

[B41] Novotny R, Pfoestl A, Messner P, Schaffer C (2004). Genetic organization of chromosomal S-layer glycan biosynthesis loci of Bacillaceae. Glycoconj J.

[B42] Schaffer C, Messner P (2004). Surface-layer glycoproteins: an example for the diversity of bacterial glycosylation with promising impacts on nanobiotechnology. Glycobiology.

[B43] Pfoestl A, Hofinger A, Kosma P, Messner P (2003). Biosynthesisof dTDP-3-acetamido-3,6-dideoxy-alpha-D-galactose in *Aneurinibacillus thermoaerophilus *L420-91T. J Biol Chem.

[B44] Eckstein TM, Belisle JT, Inamine JM (2003). Proposed pathway for the biosynthesis of serovar-specific glycopeptidolipids in *Mycobacterium avium *serovar 2. Microbiology.

[B45] Patil PB, Sonti RV (2004). Variation suggestive of horizontal gene transfer at a lipopolysaccharide (lps) biosynthetic locus in *Xanthomonas oryzae *pv. *oryzae*, the bacterial leaf blight pathogen of rice. BMC Microbiol.

[B46] Seifert GJ (2004). Nucleotide sugar interconversions and cell wall biosynthesis: how to bring the inside to the outside. Curr Opin Plant Biol.

[B47] Lopez AB, Sener K, Jarroll EL, van Keulen H (2003). Transcription regulation is demonstrated for five key enzymes in *Giardia intestinalis *cyst wall polysaccharide biosynthesis. Mol Biochem Parasitol.

[B48] Prescott AG, Lloyd MD (2000). The iron(II) and 2-oxoacid-dependent dioxygenases and their role in metabolism. Nat Prod Rep.

[B49] Baggaley KH, Brown AG, Schofield CJ (1997). Chemistry and biosynthesis of clavulanic acid and other clavams. Nat Prod Rep.

[B50] WoLF PSORT. Protein Subcellular Localization Prediction.

[B51] Andersson JO, Hirt RP, Foster PG, Roger AJ (2006). Evolution of four gene families with patchy phylogenetic distributions: influx of genes into protist genomes. BMC Evol Biol.

[B52] Andersson JO (2005). Lateral gene transfer in eukaryotes. Cell Mol Life Sci.

[B53] Kather B, Stingl K, van der Rest ME, Altendorf K, Molenaar D (2000). Another unusual type of citric acid cycle enzyme in *Helicobacter pylori*: the malate:quinone oxidoreductase. J Bacteriol.

[B54] Molenaar D, van der Rest ME, Drysch A, Yucel R (2000). Functionsof the membrane-associated and cytoplasmic malate dehydrogenases in the citric acid cycle of *Corynebacterium glutamicum*. J Bacteriol.

[B55] Van der Rest ME, Frank C, Molenaar D (2000). Functions of themembrane-associated and cytoplasmic malate dehydrogenases in the citric acid cycle of *Escherichia coli*. J Bacteriol.

[B56] Gardner MJ, Shallom SJ, Carlton JM, Salzberg SL, Nene V, Shoaibi A, Ciecko A, Lynn J, Rizzo M, Weaver B (2002). Sequence of *Plasmodium falciparum *chromosomes 2, 10, 11 and 14. Nature.

[B57] Uyemura SA, Luo S, Vieira M, Moreno SN, Docampo R (2004). Oxidative phosphorylation and rotenone-insensitive malate- and NADH-quinone oxidoreductases in *Plasmodium yoelii yoelii *mitochondria in situ. J Biol Chem.

[B58] Chen RD, Gadal P (1990). Structure, function and regulation of NAD and NADP dependent isocitrate dehydrogenase in higher plants and in other organisms. Plant Physiol Biochem.

[B59] Schnarrenberger C, Martin W (2002). Evolution of the enzymes of the citric acid cycle and the glyoxylate cycle of higher plants. A case study of endosymbiotic gene transfer. Eur J Biochem.

[B60] Lang M, Apt KE, Kroth PG (1998). Protein transport into "complex" diatom plastids utilizes two different targeting signals. J Biol Chem.

[B61] Patron NJ, Waller RF, Archibald JM, Keeling PJ (2005). Complex protein targeting to dinoflagellate plastids. J Mol Biol.

[B62] Suzuki M, Sahara T, Tsuruha J, Takada Y, Fukunaga N (1995). Differential expression in *Escherichia coli *of the *Vibrio *sp. strain ABE-1 icdI and icdII genes encoding structurally different isocitrate dehydrogenase isozymes. J Bacteriol.

[B63] Kang CH, Shin WC, Yamagata Y, Gokcen S, Ames GF, Kim SH (1991). Crystal structure of the lysine-, arginine-, ornithine-binding protein (LAO) from *Salmonella typhimurium *at 2.7-A resolution. J Biol Chem.

[B64] Jorgensen R, Carr-Schmid A, Ortiz PA, Kinzy TG, Andersen GR (2002). Purification and crystallization of the yeast elongation factor eEF2. Acta Crystallogr D Biol Crystallogr.

[B65] Moreira D, Le Guyader H, Philippe H (2000). The origin of red algae and the evolution of chloroplasts. Nature.

[B66] Regier JC, Shultz JW (2001). Elongation factor-2: a useful gene for arthropod phylogenetics. Mol Phylogenet Evol.

[B67] Kullnig-Gradinger CM, Szakacs G, Kubicek CP (2002). Phylogenyand evolution of the genus *Trichoderma*: a multigene approach. Mycol Res.

[B68] Bhattacharya D, Yoon HS, Hackett JD (2004). Chromalveolates unite: endosymbiosis connects the dots. BioEssays.

[B69] Liapounova NA, Hampl V, Gordon PM, Sensen CW, Gedamu L, Dacks JB (2006). Reconstructing the mosaic glycolytic pathway of the anaerobic eukaryote *Monocercomonoides*. Eukaryot Cell.

[B70] Andersson JO, Roger AJ (2003). Evolution of glutamate dehydrogenase genes: evidence for lateral gene transfer within and between prokaryotes and eukaryotes. BMC Evol Biol.

[B71] Andersson JO, Sarchfield SW, Roger AJ (2005). Gene transfers from nanoarchaeota to an ancestor of diplomonads and parabasalids. Mol Biol Evol.

[B72] Andersson J, Katz L, Bhattacharya D (2006). Genome evolution of anaerobic protists: metabolic adaptation via gene acquisition. Genomics and Evolution of Microbial Eukaryotes.

[B73] Hao W, Golding GB (2006). The fate of laterally transferred genes: life in the fast lane to adaptation or death. Genome Res.

[B74] Koike K, Sekiguchi H, Kobiyama A, Takishita K, Kawachi M, Koike K, Ogata T (2005). A novel type of kleptoplastidy in *Dinophysis *(Dinophyceae): presence of haptophyte-type plastid in *Dinophysis mitra*. Protist.

[B75] Gast RJ, Moran DM, Dennett MR, Caron DA (2007). Kleptoplasty in an Antarctic dinoflagellate: caught in evolutionary transition?. Environ Microbiol.

[B76] Tengs T, Dahlberg OJ, Shalchian-Tabrizi K, Klaveness D, Rudi K, Delwiche CF, Jakobsen KS (2000). Phylogenetic analyses indicate that the 19'Hexanoyloxy-fucoxanthin-containing dinoflagellates have tertiary plastids of haptophyte origin. Mol Biol Evol.

[B77] Doolittle WF (1998). You are what you eat: a gene transferratchet could account for bacterial genes in eukaryotic nuclear genomes. Trends Genet.

[B78] Graham L, Wilcox L, (eds.) (2000). Algae.

[B79] Sogayar MI, Gregorio EA (1989). Uptake of bacteria by trophozoites of *Giardia duodenalis *(Say). Ann Trop Med Parasitol.

[B80] Pereira-Neves A, Benchimol M (2007). Phagocytosis by *Trichomonas vaginalis*: new insights. Biol Cell.

[B81] Flavin M, Nerad TA (1993). *Reclinomonas americana *N. G., N. Sp., a new freshwater heterotrophic flagellate. J Eukaryot Microbiol.

[B82] Cohen CJ, Bacon R, Clarke M, Joiner K, Mellman I (1994). *Dictyostelium discoideum *mutants with conditional defects in phagocytosis. J Cell Biol.

[B83] Watkins RF, Gray MW (2006). The frequency of eubacterium-to-eukaryote lateral gene transfers shows significant cross-taxa variation within amoebozoa. J Mol Evol.

[B84] Hall C, Brachat S, Dietrich FS (2005). Contribution ofhorizontal gene transfer to the evolution of *Saccharomyces cerevisiae*. Eukaryot Cell.

[B85] Hooper SD, Berg OG (2003). Duplication is more common among laterally transferred genes than among indigenous genes. Genome Biol.

[B86] DFCI Gene Indices Software Tools. http://compbio.dfci.harvard.edu/tgi/software/.

[B87] Pertea G, Huang X, Liang F, Antonescu V, Sultana R, Karamycheva S, Lee Y, White J, Cheung F, Parvizi B (2003). TIGR Gene Indices clustering tools (TGICL): a software system for fast clustering of large EST datasets. Bioinformatics.

[B88] Emboss. http://emboss.sourceforge.net/.

[B89] Lupas N, Frickey T (2004). PhyloGenie: automated phylome generation and analysis. Nucleic Acids Res.

[B90] National Center for Biotechnology Information. http://www.ncbi.nlm.nih.gov/.

[B91] JGI. DOE Joint Genome Institute. http://www.jgi.doe.gov/.

[B92] Cyanidioschyzon merolae Genome Project. http://merolae.biol.s.u-tokyo.ac.jp/.

[B93] The *Galdieria sulphuraria *Genome Project. http://genomics.msu.edu/galdieria/.

[B94] The National Center for Biotechnology Information Basic Local Alignment Search Tool (BLAST). http://www.ncbi.nlm.nih.gov/BLAST/.

[B95] TreeView. Tree drawing software for Apple Macintoshand Windows. http://taxonomy.zoology.gla.ac.uk/rod/treeview.html.

[B96] Genoscope. The French National Sequencing Center. http://www.genoscope.cns.fr/externe/English/corps_anglais.html.

[B97] Matsuzaki M, Misumi O, Shin IT, Maruyama S, Takahara M, Miyagishima SY, Mori T, Nishida K, Yagisawa F, Nishida K (2004). Genome sequence of the ultrasmall unicellular red alga *Cyanidioschyzon merolae *10D. Nature.

[B98] Weber AP, Oesterhelt C, Gross W, Brautigam A, Imboden LA, Krassovskaya I, Linka N, Truchina J, Schneidereit J, Voll H (2004). EST-analysis of the thermo-acidophilic red microalga *Galdieria sulphuraria *reveals potential for lipid A biosynthesis and unveils the pathway of carbon export from rhodoplasts. Plant Mol Biol.

[B99] BioEdit. Biological sequence alignment editor for Windows 95/98/NT/2000/XP. http://www.mbio.ncsu.edu/BioEdit/bioedit.html.

[B100] SignalP 3.0 Server. http://www.cbs.dtu.dk/services/SignalP/.

[B101] Bendtsen JD, Nielsen H, von Heijne G, Brunak S (2004). Improved prediction of signal peptides: SignalP 3.0. J Mol Biol.

[B102] TargetP 1.1 Server. http://www.cbs.dtu.dk/services/TargetP/.

[B103] Emanuelsson O, Nielsen H, Brunak S, von Heijne G (2000). Predicting subcellular localization of proteins based on their N-terminal amino acid sequence. J Mol Biol.

[B104] MITOPROT: Prediction of mitochondrial targeting sequences. http://ihg.gsf.de/ihg/mitoprot.html.

[B105] Blast 2 Sequences. http://www.ncbi.nlm.nih.gov/blast/bl2seq/wblast2.cgi.

[B106] Guindon S, Gascuel O (2003). A simple, fast, and accurate algorithm to estimate large phylogenies by maximum likelihood. Syst Biol.

[B107] Guindon S, Lethiec F, Duroux P, Gascuel O (2005). PHYML Online – a web server for fast maximum likelihood-based phylogenetic inference. Nucleic Acids Res.

[B108] Felsenstein J (1985). Confidence limits on phylogenies: an approach using the bootstrap. Evolution.

[B109] PHYLIP V3.63. http://evolution.genetics.washington.edu/phylip.html.

[B110] Huelsenbeck JP, Ronquist F (2001). Bayesian inference of phylogenetic trees. Bioinformatics.

[B111] Bhattacharya lab downloads page. http://www.biology.uiowa.edu/debweb/downloads/.

