# Supplementary material for: Horizontal gene transfer in chromalveolates
Source: BMC Evol Biol. 2007 Sep 25;7:173. doi: 10.1186/1471-2148-7-173 (PMC2064935; doi:10.1186/1471-2148-7-173)
Supplement: Additional file 1 — Supporting materials for the study. The complete list of sequences used in this study is shown as well as three figures that show the origins of iron-containing alcohol dehydrogenase (Fe-ADH) and NAD-dependent aldehyde dehydrogenase (PutA) in eukaryotes by multiple HGTs, the origins of substrate-bound periplasmic binding protein (PBPb) and silent information regulator 2 (SIR2) in protists, and examples of HGT events from bacteria to dinoflagellates. [file 1471-2148-7-173-S1.pdf]

Additional File

Title: Supporting materials for the study.

Sequences used in this study.

Protein	Taxon	Sequence Accession Number
**WECE**	*Seculamonas ecuadoriensis*	EC816654; EC816654
	Jakoba bahamiensis	EC686674, EC685007, EC684822
	Sawyeria marylandensis	EC826207
**MVIM**	*Karlodinium micrum*	EC160173
	Jakoba bahamiensis	EC687464, EC685628
	Sawyeria marylandensis	EC825128
**Epimerase**	*Phytophthora ramorum*	jgi|Phyra1_1|73107|fgenesh1_pg.C_scaffold_2000172*
	Thalassiosira pseudonana	jgi|Thaps3|35046|e_gw1.6.331.1*
	Giardia lamblia	XP_769114, AAO39053
	Spironucleus barkhanus	ABI15605
**CAS -like**	*Karlodinium micrum*	EC156868, EC158513, EC148099, EC160669
	Alexandrium tamarense	CK784871
	Emiliania huxleyi	CF753752
	Isochrysis galbana	EC140831
	Chlamydomonas reinhardtii	jgi|Chlre3|127464|estExt_gwp_1H.C_20095*
	Ostreococcus lucimarinus	jgi|Ostta4|35883|1200010181*
	Ostreococcus tauri	jgi|Ost9901_3|26795|eugene.1200010140*
**MQO-DH**	*Karlodinium micrum*	EC161113, EC156079, EC151820
	Alexandrium tamarense	CF948531, CV554334
	Heterocapsa triquetra	DT382779
	Emiliania huxleyi	CX779033
**MQO-A**	*Plasmodium yoelii*	EAA17232
	Plasmodium berghei	CAH94470
	Plasmodium chabaudi	XP_742339
	Plasmodium falciparum	CAG25406
	Toxoplasma gondii	ABE76504
	Eimeria tenella	CAK51433
**mtMDH**	*Karlodinium micrum*	EC154776
	Alexandrium tamarense	CK784465
	Isochrysis galbana	EC147060, EC147055
**cytMDH**	*Isochrysis galbana*	EC143180, EC146073
	Pavlova lutheri	EC176556, EC176389, EC176299
**NADP-IDH**	*Karlodinium micrum*	EC155818, EC150415
	Alexandrium tamarense	CK433031, CK784788, CK783806
	Amphidinium carterae	CF065460, CF064816
	Heterocapsa triquetra	DT385947, DT385631

	Lingulodinium polyedrum	BP743001, CD809840
	Phaeodactylum tricornutum	jgi|Phatr2|34602|fgenesh1_pg.C_chr_5000402*, jgi|Phatr2|45017|estExt_fgenesh1_pg.C_chr_50402*
	Thalassiosira pseudonana	jgi|Thaps3|1456|fgenesh1_pg.C_chr_1000590*
	Isochrysis galbana	EC138409
**FE-ADH**	*Naegleria grubery*	jgi|Naegr1|56035|estExt_fgeneshHS_kg.C_70004*
**PutA**	*Karlodinium micrum*	EC155616
	Phaeodactylum tricornutum	jgi|Phatr2|21068|estExt_gwp_gw1.C_chr_110150*
	Phytophthora ramorum	jgi|Phyra1_1|71205|fgenesh1_pm.C_scaffold_8000011*
	Jakoba libera	EC693135
	Seculamonas ecuadoriensis	EC816325
	Physarum polycephalum	EC757810
	Cyanidioschyzon merolae	gnl|CMER|CMR066C**
	Chlamydomonas reinhardtii	jgi|Chlre3|24394|estExt_fgenesh1_pm.C_440003*
	Ostreococcus tauri	jgi|Ostta4|28991|estExt_gwp_GeneWisePlus.C_Chr_10.00010194*
**PBPb**	*Karlodinium micrum*	EC150730
	Alexandrium tamarense	CF948337
	Emiliania huxleyi	EG034291
	Isochrysis galbana	EC139997
	Phaeodactylum tricornutum	jgi|Phatr2|34826|fgenesh1_pg.C_chr_6000199*
	Euglena gracilis	EC676369
**SIR2**	*Sawyeria marylandensis*	EC825857
	Acanthamoeba castellanii	EC105362
	Phaeodactylum tricornutum	jgi|Phatr2|36942|fgenesh1_pg.C_chr_11000295*
	Thalassiosira pseudonana	jgi|Thaps3|269475|estExt_thaps1_ua_kg.C_chr_100004*
	Chlamydomonas reinhardtii	jgi|Chlre3|113408|e_gwW.4.63.1
**AslA**	*Alexandrium tamarense*	CK785860
**ATS1**	*Karlodinium micrum*	EC159487, EC155725, EC159597, EC159682, EC158675
	Amphidinium carterae	CF065437
	Lingulodinium polyedrum	BP742850
**COG3618**	*Alexandrium tamarense*	EC154481
	Karlodinium micrum	CK431462
**COG3022**	*Alexandrium tamarense*	CK785470
	Lingulodinium polyedrum	BP743223
**EF2**	*Karlodinium micrum*	EC160362, EC159484, EC152658
	Alexandrium tamarense	CK782463
	Amphidinium carterae	CF065997, CF067005
	Heterocapsa triquetra	DT385971, DT385641
	Lingulodinium polyedrum	BP743144
	Pyrocystis lunula	BU582439
	Paramecium tetraurelia	scaffold_151
	Isochrysis galbana	EC145862, EC143408, EC143130

	Pavlova lutheri	EC177146, EC177552, EC176343
	Phytophthora ramorum	jgi|Phyra1_1|71206|fgenesh1_pm.C_scaffold_8000012*
	Phytophthora sojae	jgi|Physo1_1|109259|estExt_fgenesh1_pm.C_810006*
	Thalassiosira pseudonana	jgi|Thaps3|269148|estExt_thaps1_ua_kg.C_chr_60290*
	Jakoba libera	EC692709, EC692770, EC690987
	Cyanophora paradoxa	EC660009, EG946313, EG944985, EG946356
	Glaucocystis nostochinearum	EC124674

Note: All sequences except for the sequences labeled with "*" and "**" are available from the GenBank nonredundant data base and dbEST (http://www.ncbi.nlm.nih.gov/). Sequences labeled with "*"are available from the DOE Joint Genome Institute (JGI) (http://www.jgi.doe.gov/); "**" – from the *Cyanidioschyzon merolae* Genome Project database (http://merolae.biol.s.u-tokyo.ac.jp/

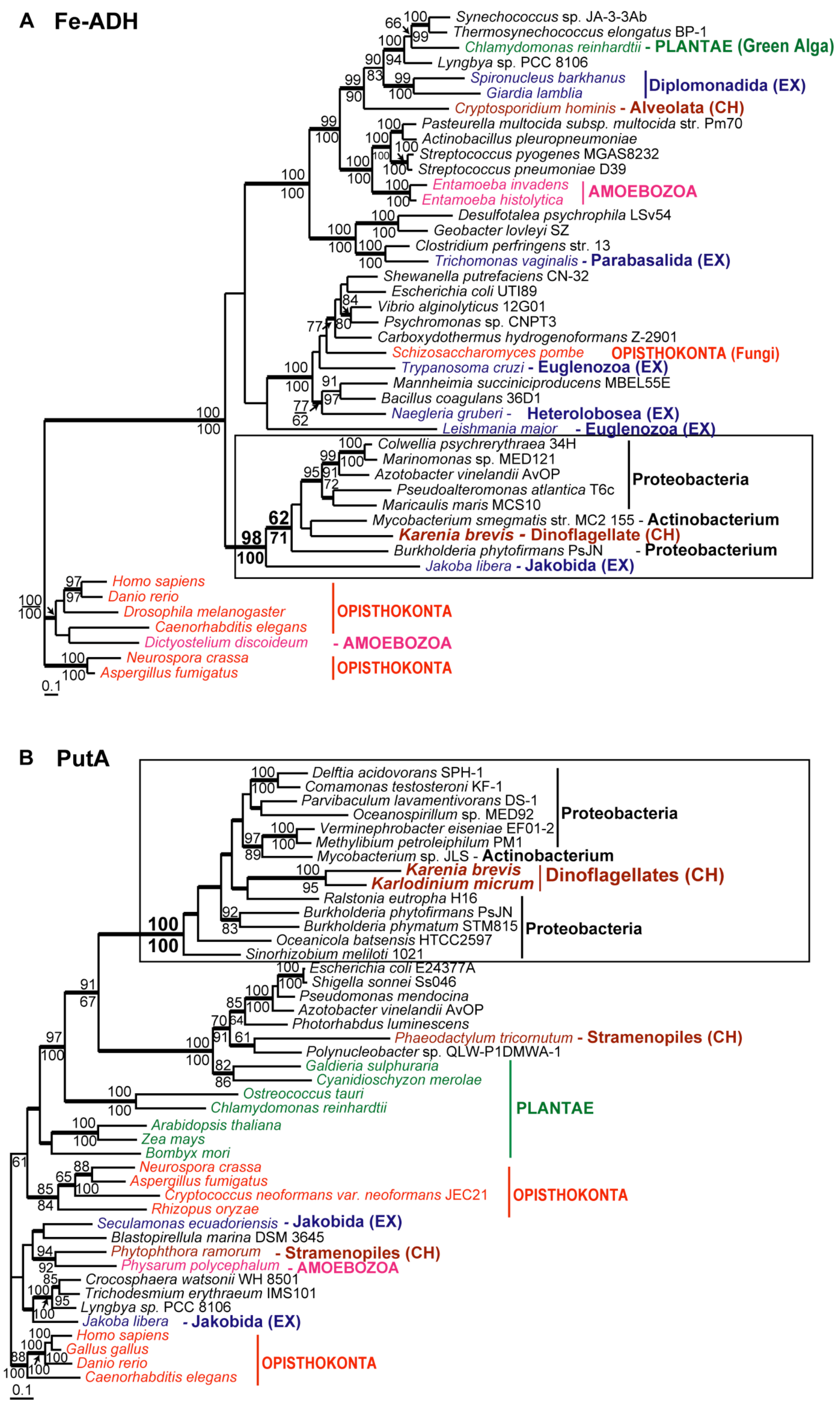

Origins of iron-containing alcohol dehydrogenase (Fe-ADH) and NAD-dependent aldehyde dehydrogenase (PutA) in eukaryotes by multiple HGTs

A. ML tree of Fe-ADH. **B.** ML tree of PutA. The numbers above and below the branches are the results of ML and NJ bootstrap analyses, respectively. Only bootstrap values ≥ 60% are shown. The thick branches indicate ≥ 0.96 posterior probability from Bayesian inference. Branch lengths are proportional to the number of substitutions per site (see scale bars). CH and brawn color indicate a supergroup Chromalveolata; EX and blue color indicate a supergroup Excavata. Red color indicates Opisthokonta, pink – Amoebozoa, green – Plantae.

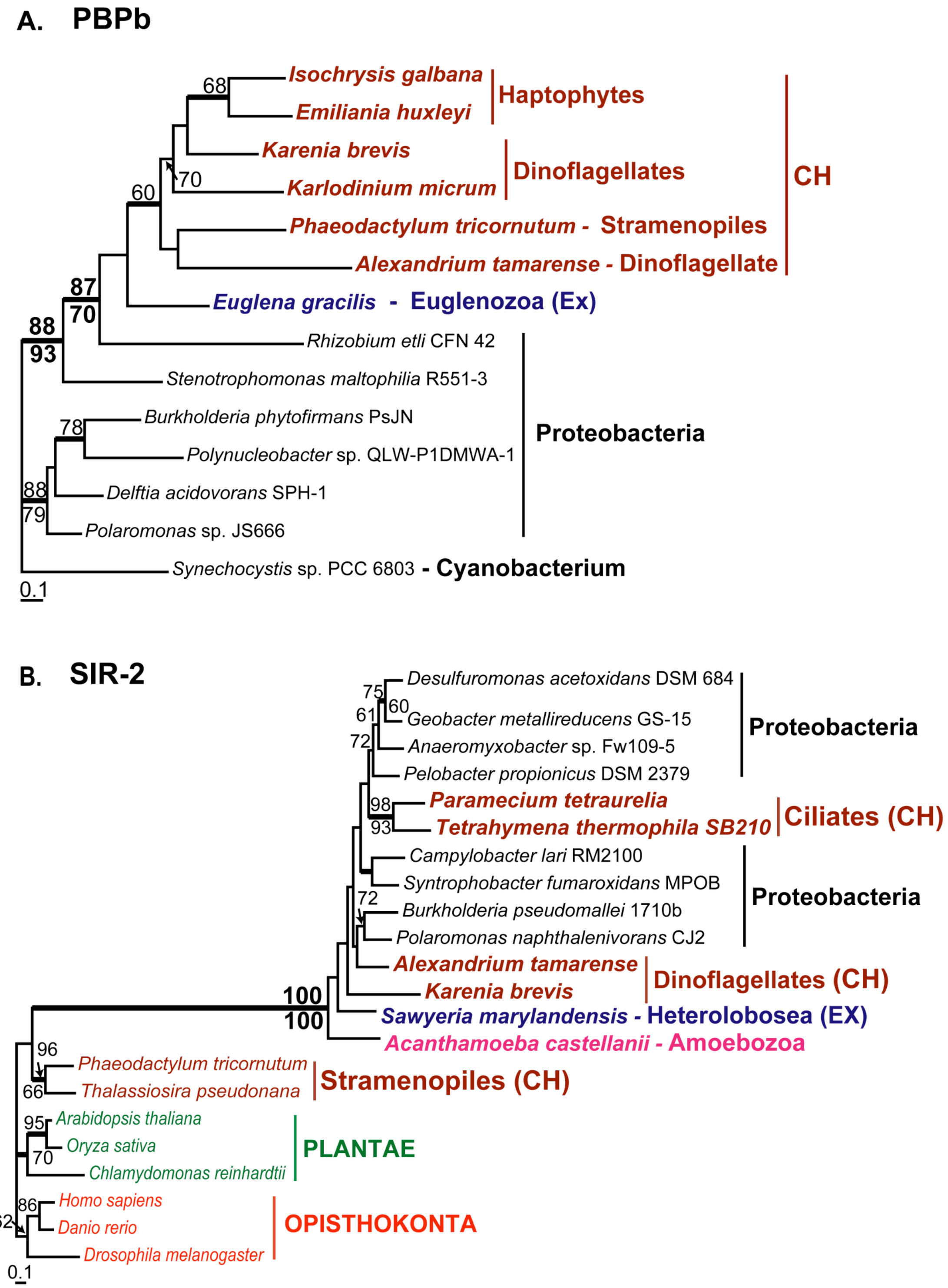

Origins of substrate-bound periplasmic binding protein (PBPb) and silent information regulator 2 (SIR2) in protists

A. ML tree of PBPb. **B.** ML tree of SIR2. The numbers above and below the branches are the results of ML and NJ bootstrap analyses, respectively. Only bootstrap values ≥ 60% are shown. The thick branches indicate ≥ 0.96 posterior probability from Bayesian inference. Branch lengths are proportional to the number of substitutions per site (see scale bars). Short abbreviations and the color scheme are as above.

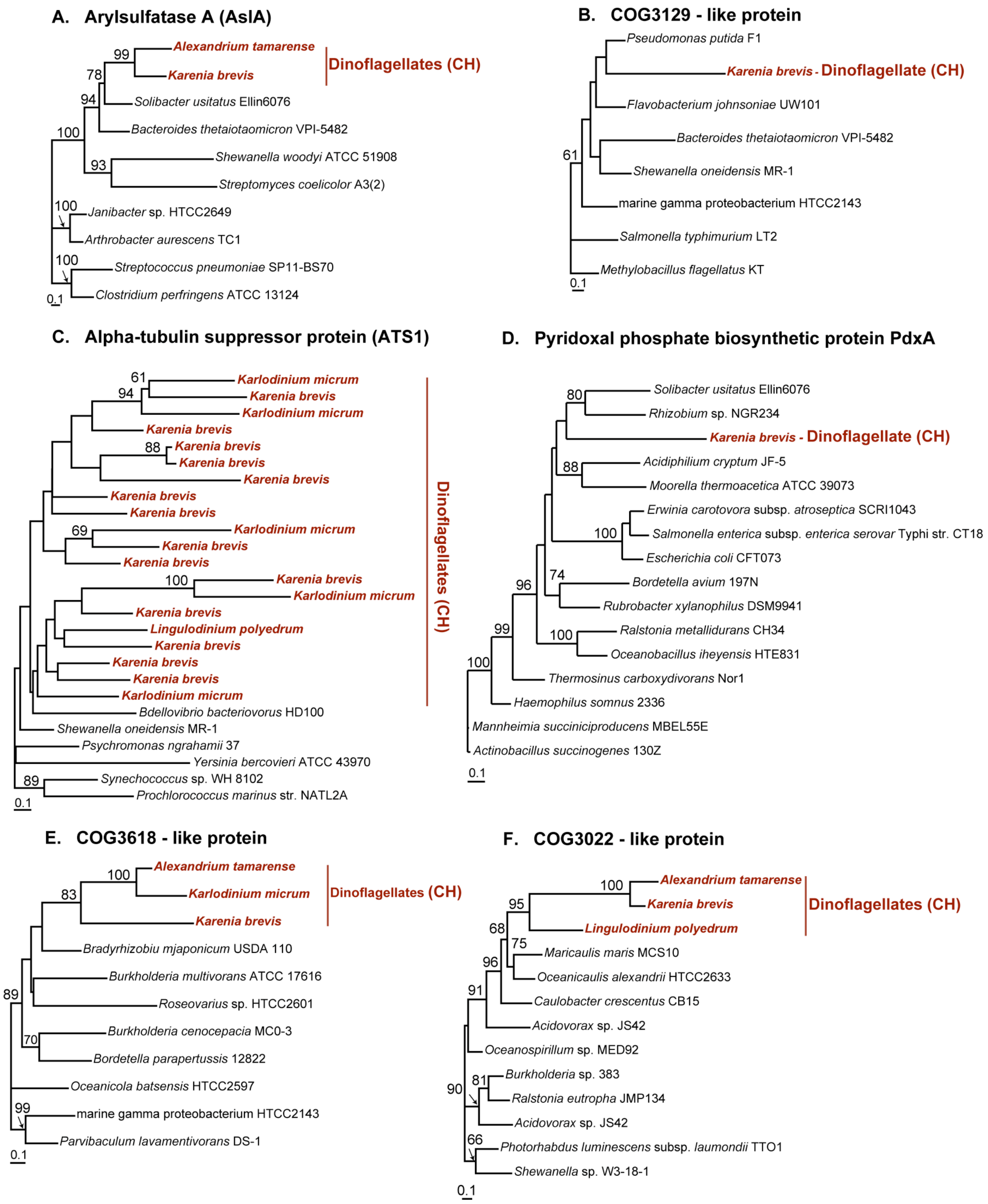

Horizontal gene transfer from bacteria to dinoflagellates

ML trees of: **A.** Arylsulfatase A (AslA). **B.** COG3129-like protein. **C.** Alpha-tubulin suppressor protein (ATS1). **D.** Pyridoxal phosphate biosynthetic protein PdxA. **E.** COG3618-like protein. **F.** COG3022-like protein. The numbers above the branches are the results of ML bootstrap analyses. Only bootstrap values ≥ 60% are shown. Branch lengths are proportional to the number of substitutions per site (see scale bars). Short abbreviations and the color scheme are as above.
